# Supplementary material for: Development and validation of a casemix classification to predict costs of specialist palliative care provision across inpatient hospice, hospital and community settings in the UK: a study protocol
Source: BMJ Open. 2018 Mar 17;8(3):e020071. doi: 10.1136/bmjopen-2017-020071 (PMC5879599; doi:10.1136/bmjopen-2017-020071)
Supplement: Author's manuscript [file bmjopen-2017-020071.draft_revisions.pdf]

BMJ Open

BMJ Open is committed to open peer review. As part of this commitment we make the peer review history of every article we publish publicly available.

When an article is published we post the peer reviewers' comments and the authors' responses online. We also post the versions of the paper that were used during peer review. These are the versions that the peer review comments apply to.

The versions of the paper that follow are the versions that were submitted during the peer review process. They are not the versions of record or the final published versions. They should not be cited or distributed as the published version of this manuscript.

BMJ Open is an open access journal and the full, final, typeset and author-corrected version of record of the manuscript is available on our site with no access controls, subscription charges or pay-per-view fees (http://bmjopen.bmj.com).

If you have any questions on BMJ Open's open peer review process please email info.bmjopen@bmj.com

Development and validation of a casemix classification to predict costs of specialist palliative care provision across inpatient hospice, hospital, and community settings in the UK: A study protocol

Journal:	*BMJ Open*
Manuscript ID	bmjopen-2017-020071
Article Type:	Protocol
Date Submitted by the Author:	12-Oct-2017

Complete List of Authors:	Guo, Ping; King's College London, Cicely Saunders Institute, Department of Palliative Care, Policy and Rehabilitation<br>Dzingina, Mendwas; King's College London, Cicely Saunders Institute, Department of Palliative Care, Policy and Rehabilitation<br>Firth, Alice; King's College London, Cicely Saunders Institute, Department of Palliative Care, Policy and Rehabilitation<br>Davies, Joanna; King's College London, Cicely Saunders Institute, Department of Palliative Care, Policy and Rehabilitation<br>Douiri, Abdel; King's College London, Primary Care And Public Health Sciences<br>O'Brien, Suzanne; King's College London, Cicely Saunders Institute, Department of Palliative Care, Policy and Rehabilitation<br>Pinto, Cathryn; King's College London, Cicely Saunders Institute, Department of Palliative Care, Policy and Rehabilitation<br>Pask, Sophie; King's College London, Cicely Saunders Institute, Department of Palliative Care, Policy and Rehabilitation<br>Higginson, Irene; Kings College London, Cicely Saunders Institute, Department of Palliative Care, Policy and Rehabilitation<br>Eagar, Kathy; University of Wollongong, Australian Health Services Research Institute, Centre for Health Service Development<br>Murtagh, Fliss; University of Hull, Wolfson Palliative Care Research Centre, Hull York Medical School

Keywords:	Adult palliative care < PALLIATIVE CARE, Casemix classification, Validation, Cost predictors

SCHOLARONE™
Manuscripts

TITLE PAGE

Full title:

Development and validation of a casemix classification to predict costs of specialist palliative care provision across inpatient hospice, hospital, and community settings in the UK: A study protocol

Author details:

Dr Ping Guo,
Project Manager and Research Associate
Cicely Saunders Institute, Department of Palliative Care, Policy & Rehabilitation, King's College London, Bessemer Road, SE5 9PJ, UK
Correspondence to P. Guo: Email: ping.guo@kcl.ac.uk Telephone: 020 7848 5523

Dr Mendwas Dzingina, Cicely Saunders Institute, Department of Palliative Care, Policy & Rehabilitation, King's College London, Bessemer Road, SE5 9PJ, UK

Alice M Firth, Cicely Saunders Institute, Department of Palliative Care, Policy & Rehabilitation, King's College London, Bessemer Road, SE5 9PJ, UK

Joanna M Davies, Cicely Saunders Institute, Department of Palliative Care, Policy & Rehabilitation, King's College London, Bessemer Road, SE5 9PJ, UK

Dr Abdel Douiri, Department of Primary Care and Public Health Sciences, Addison House, Guy's Campus, King's College London, UK

Suzanne M O'Brien, Cicely Saunders Institute, Department of Palliative Care, Policy & Rehabilitation, King's College London, Bessemer Road, SE5 9PJ, UK

Cathryn Pinto, Cicely Saunders Institute, Department of Palliative Care, Policy & Rehabilitation, King's College London, Bessemer Road, SE5 9PJ, UK

Sophie Pask, Cicely Saunders Institute, Department of Palliative Care, Policy & Rehabilitation, King's College London, Bessemer Road, SE5 9PJ, UK

Professor Irene J Higginson, Cicely Saunders Institute, Department of Palliative Care, Policy & Rehabilitation, King's College London, Bessemer Road, SE5 9PJ, UK

Professor Kathy Eagar, University of Wollongong, Australian health Services Research Institute, Centre for Health Service Development, Wollongong, Australia

Professor Fliss EM Murtagh, Wolfson Palliative Care Research Centre, Hull York Medical School, University of Hull, UK

For peer review only - http://bmjopen.bmj.com/site/about/guidelines.xhtml

Development and validation of a casemix classification to predict costs of specialist palliative care provision across inpatient hospice, hospital, and community settings in the UK: A study protocol

ABSTRACT

Introduction: Provision of palliative care is inequitable with wide variations across conditions and settings in the UK. Lack of a standard way to classify by case complexity is one of the principle obstacles to addressing this. We aim to develop and validate a casemix classification to support the prediction of costs of specialist palliative care provision.

Methods and analysis:

Phase I - A cohort study to determine the variables and potential classes to be included in a casemix classification. Data are collected from clinicians in palliative care services across inpatient hospice, hospital, and community settings on: patient demographics, potential complexity/casemix criteria, and patient-level resource use. Cost predictors are derived using multivariate regression and then incorporated into a classification using Classification and Regression Trees (CART). Internal validation will be conducted by bootstrapping to quantify any optimism in the predictive performance (calibration and discrimination) of the developed classification.

Phase II - A mixed methods cohort study across settings for external validation of the classification developed in Phase I. Patient and family caregiver data will be collected longitudinally on demographics, potential complexity/casemix criteria, and patient-level resource use. This will be triangulated with data collected from clinicians on potential complexity/casemix criteria and patient-level resource use, and with qualitative interviews with patients and caregivers about care provision across difference settings. The classification will be refined on the basis of its performance in the validation data set.

Ethics and dissemination: The study has been approved by the National Health Service (NHS) Health Research Authority Research Ethics Committee. The results are expected to be published in 2018.

Study registration number: ISRCTN90752212

For peer review only - http://bmjopen.bmj.com/site/about/guidelines.xhtml

Strengths and limitations of this study

- This is the first study in the UK to determine the variables and potential classes to be included in a casemix classification, which predicts costs of specialist palliative care provision across inpatient hospice, hospital, and community settings.
- Transparent reporting of a multivariable prediction model for individual prognosis or diagnosis (TRIPOD) is used to guide the reporting of this study.
- The newly developed casemix classification in phase I will be externally validated by using a different dataset in phase II.
- This study also promotes the implementation of outcome measures in palliative care into routine clinical practice across different settings of care.
- This is a UK focused study so the casemix classification may not be directly applied to palliative care in other countries without further investigation and refinement.

For peer review only

For peer review only - http://bmjopen.bmj.com/site/about/guidelines.xhtml

INTRODUCTION

People with advanced and incurable illness often suffer complex and multiple symptoms and psychosocial concerns because of their illness or impending death.[1, 2] Their families may provide day-to-day care, as well as being affected by their own anxieties, concerns, and potential losses. These bring increased need for health and social care, with need being defined as 'the ability to benefit from health or social care interventions'.[3] Palliative care has developed to meet the needs of these patients and families, which addresses physical/psychological symptoms and provides social, practical and spiritual support. The UK ranks first in the 2015 Quality of Death Index - a measure of the quality of dying in 80 countries,[4] and the hospice movement in the UK has provided a model of good palliative care for those in need.

However, marked inequities exist in provision of palliative care across England. Older people and those with non-cancer diagnoses are less likely to access specialist palliative care.[5, 6] There are also major geographical variations, ranging from £186 to £6,213 per person across different Primary Care Trusts in 2010,[7, 8] often resulting in a poor match between individual needs, resources provided to meet those needs, and health outcomes achieved. With an ageing population and increasing rates of chronic diseases, the growing healthcare burden is overwhelmingly challenging in terms of health resource allocation around the UK.[9] With recognition of the constraints on resources, there is support for the systematic approach to mapping individual needs accurately and improving the quality and efficiency of palliative care. This has been endorsed as a high priority nationally.[7]

The Diagnosis Related Group is a useful classification of healthcare needs driven by the diagnosis but is inappropriate for palliative care, because palliative care needs are not driven by the diagnosis but by factors such as functional status and symptoms. Palliative care needs a consistent method of classifying types of patients with complexity of needs, treatment, and costs, using casemix criteria.[10-12] It is necessary to identify those with more complex palliative needs, requiring more resources. An Australian casemix classification for palliative care was developed in 1997, empirically tested and progressively refined over time.[13-15] Its implementation proved the possibility of consistently and routinely collecting data in practice in Australia.[16] However, due to variations in outcome measures collected and palliative care provided between countries, it is unclear whether any existing palliative care classification can be easily applied to the UK to address unmet needs and resolve the inequity.

This study is part of a five year NIHR funded C-CHANGE programme (RP-PG-1210-12015). It aims to develop and validate a casemix classification for palliative care in the UK. Specific objectives are:

For peer review only - http://bmjopen.bmj.com/site/about/guidelines.xhtml

- To determine the cost predictors of specialist palliative care, adjusting for important confounding factors including unmet needs.
- To develop and validate a casemix classification for specialist palliative care.

METHODS AND ANALYSIS

The Transparent Reporting of a multivariable prediction model for Individual Prognosis Or Diagnosis (TRIPOD) Statement [17] is used to guide the reporting of this study protocol.

Study design and source of data

This study consists of two phases: Development phase (Phase I) and validation phase (Phase II). In phase I, we will identify variables that predict costs to be included in casemix classification through a cohort study with only clinician data, and develop potential casemix classes for palliative care which will be tested in individual episodes of care (internal validation). In phase II, we will prospectively validate these potential classes in a new cohort study with patient, caregiver, and clinician data, and include qualitative interviews to ensure it works during care transitions and longitudinally over the course of illness (external validation).

Phase I – Development phase

This is a cohort study to determine the variables and potential classes to be included in a casemix classification, based on individual episodes of care across inpatient (hospice and hospital) and community settings. An 'episode of care' starts when a patient is admitted to inpatient services or begins to receive specialist palliative care from a community-based or outpatient service, and ends when a patient is discharged from that service or dies. The median duration of an episode of care is expected to be under 14 days in inpatient and at a median of 72 days in community settings.[18]

Phase I was conducted between 31st July 2015 and 30th September 2016 and follow-up ended 30th November 2016. Data were collected from clinicians through surveys, including demographic/clinical data, episode start/end data, patient attributes that predict palliative care resource consumption (e.g., phase of illness, functional status, and problem severity as identified in the Australian casemix classification study [13, 14, 16]), plus information on patient-level resource use (staff activity and clinical services) in specialist palliative care settings.

Phase II – Validation phase

This phase is a mixed methods cohort study with a concurrent nested design:[19]

For peer review only - http://bmjopen.bmj.com/site/about/guidelines.xhtml

- Quantitative main component - Prospectively collect data from patients, caregivers and clinicians on palliative care needs, concerns, outcomes, and resources use.
- Qualitative nested component - Longitudinal interviews with a subsample of participants to understand care provision in each setting and transitions between settings.

Phase II will be conducted between 1st November 2016 and 31st December 2017, with follow-up ending on 28th February 2018. The same variables and measurements as Phase I will be collected from clinicians. Additionally, patient/caregiver participants will provide data on symptoms/concerns, experience of care, and their use of services, and be followed through all episodes of care from recruitment to death or the end of this study. A post bereavement survey will be conducted with caregivers where appropriate to identify symptoms/concerns immediately prior to death and support needs after death.

Participants

Phase I – Development phase

All adults (≥18 years) receiving specialist palliative care in participating sites were included, regardless of primary diagnosis. Written informed consent was taken by a qualified member of the research or clinical team. This phase included individuals with limited, fluctuating, diminishing or lack of capacity, vital to ensure that any casemix classification is applicable to all palliative patients, not just those able to consent. It is recognised that a high proportion of those have impaired capacity, and these patients may need palliative care and resources most.[20-23] Therefore, if the clinician assessed that the patient did not currently have the capacity to give consent, assent was sought from an accompanying family member, or failing that, a staff member to whom the patient was known. Where a formally appointed Power of Attorney existed, this took precedence.

Phase II – Validation phase

All adults (≥18 years) receiving specialist palliative care from 14 sites will be eligible, regardless of primary diagnosis. Written informed consent will be sought from eligible patients (and their family caregivers) at the outset, including advance consent for study follow-up if capacity is lost. This study is of minimal risk with participants not exposed to undue harm.[24]

Since transition of care is important for casemix, patient participants who transfer between settings (and caregiver participants) will be followed up. We will verbally confirm continuing consent with each participant when making such contact. If capacity of patient participants is lost, we will seek

For peer review only - http://bmjopen.bmj.com/site/about/guidelines.xhtml

advice from a personal consultee on whether the patient should remain in the study to verify no change of decisions has been expressed by the patient prior to loss of capacity. On recruitment, participants will be asked if willing to be interviewed at a later stage. We will purposively select 20-25 patients and family caregivers with at least two transitions of care for face-to-face interviews. Each semi-structured interview will last 40 minutes, but will be guided by each participant. In order to provide information on how care transitions might be better negotiated to improve outcomes and experiences, the interviews will cover communication, coordination of care, information/support needs, discharge planning, and experience of transitions.

Outcome

The outcome is cost of specialist palliative care (including per diem cost, per phase cost, and total episode cost) captured by: (1) staff activity matrix in both the development and validation phase, (2) the Palliative care Resource Use Score (PRUS) in both phases, and (3) Palliative Client Services Receipt Inventory (Pall-CSRI) in validation phase only.

Staff activity

At every contact, nurses, doctors, and allied health professionals will record the time spent on face-to-face and phone contacts, and patient level administrative time per shift using the staff activity matrix (Table 1).

Table 1 Staff activity matrix (Each box is completed in units of 5 minutes, from 0 up to 120 minutes)

Staff time (mins)	Patient	Family/carer	Professional (internal)	Professional (external)
Face-to-face/phone time				
Administrative time				

Palliative care Resource Use Score (PRUS)

PRUS will be collected by staff at change of 'Phase of Illness'[25, 26] and at the end of each episode by recording the following information for the phase which has ended:

- Level of professional input (e.g., registered nurses, specialist palliative nursing staff, palliative doctors, and social workers) and whether this met patient/family needs.
- Level of 'out of hours' services by professional designation.
- Equipment, high cost drugs, diagnostic tests, and medical imaging.
- Any unmet needs which provide insight into any gaps between needs and provision.[27-29]

For peer review only - http://bmjopen.bmj.com/site/about/guidelines.xhtml

Palliative Client Services Receipt Inventory (Pall-CSRI)

The Pall-CSRI is a patient/caregiver completed inventory of palliative care services received, and adapted from those used with palliative care populations.[30] It takes approximately 20 minutes to complete and collects retrospective information about the use of health/social care services, medication, living situation, income, employment and benefits, plus informal care. The Pall-CSRI will be collected three monthly or at the end of each episode of care whichever is earlier in the validation phase.

In the development phase, only direct care costs will be calculated but not productivity losses.[31] In the validation phase, informal care costs are considered to be in-scope, hypothesising that informal care costs are (1) greater in those with unmet needs, and (2) greater in those with non-cancer conditions. The costs of each resource item will be calculated by combining the resource use data with appropriate unit cost obtained from recognised sources including the annual compendium produced by the Personal Social Services Research Unit (PSSRU),[32] prices on the NHS supply chain website http://www.supplychain.nhs.uk, British national formulary,[33] and NHS reference costs.[34] The focus will be staff time - the main resource in palliative care documented in a variety of settings and countries.[35-37] We will attribute costs according to a standard costing methodology adopted from the current NHS costing principles.[38]

Predictors (casemix criteria)

Proposed cost predictors can be categorised into three groups: (1) predictors collected from clinicians, (2) predictors collected from patients/family caregivers, and (3) model of care.

Predictors collected from clinicians

In both the development and validation phases, clinicians will record demographic, clinical and episode start administrative data. Episode end data will be collected at the end of each episode of care. Casemix data include phase of illness (stable, unstable, deteriorating, dying), functional status (measured by Australia-modified Karnofsky Performance Status - AKPS), dependency (Modified Barthel Index), problem severity (Integrated Palliative care Outcome Scale - IPOS staff version), and caregiver needs (Table 2). Phase of illness will be assessed daily for people receiving inpatient care and at each face-to-face contact basis for those receiving community-based care. Other variables including AKPS, Modified Barthel Index, IPOS-staff (and caregiver needs when feasible) will be collected at the start of episode, end of episode, and at change of phase of illness.

For peer review only - http://bmjopen.bmj.com/site/about/guidelines.xhtml

Table 2 Data from clinicians in both development phase and validation phase

Type of data	Proposed cost predictors
Demographic data	Age
	Gender
	Postcode
	Ethnicity
	Marital status
	Living circumstances
	Need for interpreter
	Setting of care
Clinical data	Primary diagnosis
	Secondary diagnoses
	Co-morbidities
Episode start and end data	Episode start date
	Episode end date
	Endpoint of episode (discharged or died).
	Discharge destination, if discharged
Key casemix	Phase of illness
	Functional status (AKPS)
	Dependency (Modified Barthel Index)
	Problem severity (IPOS – staff version)
	Family/caregiver needs

For peer review only

Predictors collected from patients and family caregivers

Variables (Table 3) will be collected in the validation phase from patients/caregivers using face-to-face/telephone contacts and postal questionnaires according to their preferences. IPOS - patient version and Distress Thermometer will be collected from patients at the start of episode, at change of phase of illness, and at the end of episode. Views on Care (VoC) and SF-12v2 will be collected at the start and end of each episode of care. Selected questions capturing experiences of integrated care[39] will be collected at the end of episode only.

All caregiver reported measures will be collected at the start of episode, at change of phase of illness, and at the end of episode. Where phase length exceeds 4 weeks, data collected from patients (and

For peer review only - http://bmjopen.bmj.com/site/about/guidelines.xhtml

caregivers) will be captured as a change of phase. If the patient is transferred to an out of scope inpatient setting, we will follow up with the patient when he or she gets home and retrospectively collect minimum information about this episode of care.

Table 3 Predictors collected from patients and family caregivers in validation phase only

**Data collection from patients**	**Data collection from family caregivers**
IPOS – patient version	Basic demographic information
Distress Thermometer	Distress Thermometer
Views on Care (VoC)	2 caregiver questions
SF-12v2*	Zarit 6 items
Patient experiences of integrated care	

* The measure could be completed by a family caregiver if the patient is too unwell to complete.

If the patient is deceased, data such as SF-12v2 and Pall-CSRI will be collected from the participating caregiver three months after death by postal questionnaire, along with the bereavement support information and contacts. The time between death and the postal questionnaire may influence caregivers' willingness to share their views; shorter periods possibly being too upsetting to contemplate involvement.[40] There is mixed evidence of the best time to make contact with potential participants for follow-back surveys[41, 42] and 3-4 months post bereavement is considered acceptable by bereaved families.[30]

Model of care

Models of care may be a stronger cost driver than patient-level variables.[13, 14, 16] Specialist palliative care services have various configurations of staff, interventions, and other characteristics. Currently, there is no consistent way to define models of palliative care. A separate, parallel study will be conducted in which service-level data including the numbers, disciplines, and grading of staff, the nature/duration of their involvement, and use of volunteers will be collected to comprehensively characterise different models of palliative care provision in the participating sites. With findings from this step, a new categorical variable will be created representing 'model of care' to be included in our analysis.

Sample size

In the development phase, based on standard recommendations for fitting multivariate models, a minimum of 50+8×m (where m is the number of predictors) is required to test the hypothesis that

For peer review only - http://bmjopen.bmj.com/site/about/guidelines.xhtml

the population multiple correlation equals zero with a power of 80%, alpha = 5% and a medium effect size for the regression analysis (R2=0.13).[43] We estimated a sample size of 450 episodes per setting (allowing for 25% incomplete episodes and up to 10% of complete episodes being cost outliers with unusually high or low costs). A total of 1,350 patient episodes across settings is needed.

In the validation phase, the 10 events per variable (EPV) rules is used,[17] but should be data driven[44] as it can result in small sample sizes leading to overfitting and optimism. We expect the final casemix classification to contain 5-8 casemix variables based on the Australian experience.[13] Sample size is therefore estimated initially on a minimum of 10 participants per variable (50-80 participants). We have made the conservative estimate that data from 80 participants per setting (hospice, hospital, community) are needed. Allowing for 35% attrition, we estimate needing a total of 325 patients, which will provide about 1,000 patient episodes (an average of three episodes of care per patient[15]). The sample size will be re-calculated and inflated using the design effect based on intraclass correlation which will be estimated through interim analyses.

Missing data and data handling

Data will be collected prospectively and recorded on an electronic database. Any data transferred from the sites to the central study database will be carried out under the NHS Code of Practice on Confidentiality. Data will be cleaned and cross-checked using a number of internal checks (automated data validation on entry, independent cross-checking of a 5% sample of data from each site, and range checking across all data types) to track errors and inconsistencies and amend where possible. Checked data will be transferred to statistical software (Stata SE v12) for analysis. Missing Value Analysis will be used to quantify missing values and understand the reason for missing data: drop-out of participants, errors in data entry, and missing with no identifiable reasons. We will adopt multiple imputation technique based on Markov Chain Monte Carlo (MCMC) techniques if the percentage of observed missing values in independent variables exceeds 10% provided values are missing at random, and carry out sensitivity analysis for effects on casemix classification, predictive validity, and misclassification, where feasible.

Statistical analysis methods

We will use descriptive statistics including frequencies, mean and standard deviation; and median and interquartile ranges as appropriate to describe the sample characteristics, and number and length of episodes of care. Trajectory of outcomes and costs will be described, comparing patterns in cancer and non-cancer patients, and contrasting those with no unmet needs and those with unmet

For peer review only - http://bmjopen.bmj.com/site/about/guidelines.xhtml

needs. We will compare our data with existing phase and episode data from the Australian Palliative Care Outcome Collaboration.[45] In the Australian classification, five casemix criteria (phase of illness, problem severity, functional status and dependency, age, model of care) were found to be most strongly predictive of resource use. If our analyses demonstrate the same five criteria as most predictive of resource use, the Australian classification or a refinement of it will be adopted. If there is disparity, then the data on individual casemix criteria will be used to develop a new casemix classification, using a recursive partitioning approach – specifically classification and regression trees (CART)[46, 47] compared to multivariable regression (Figure 1).

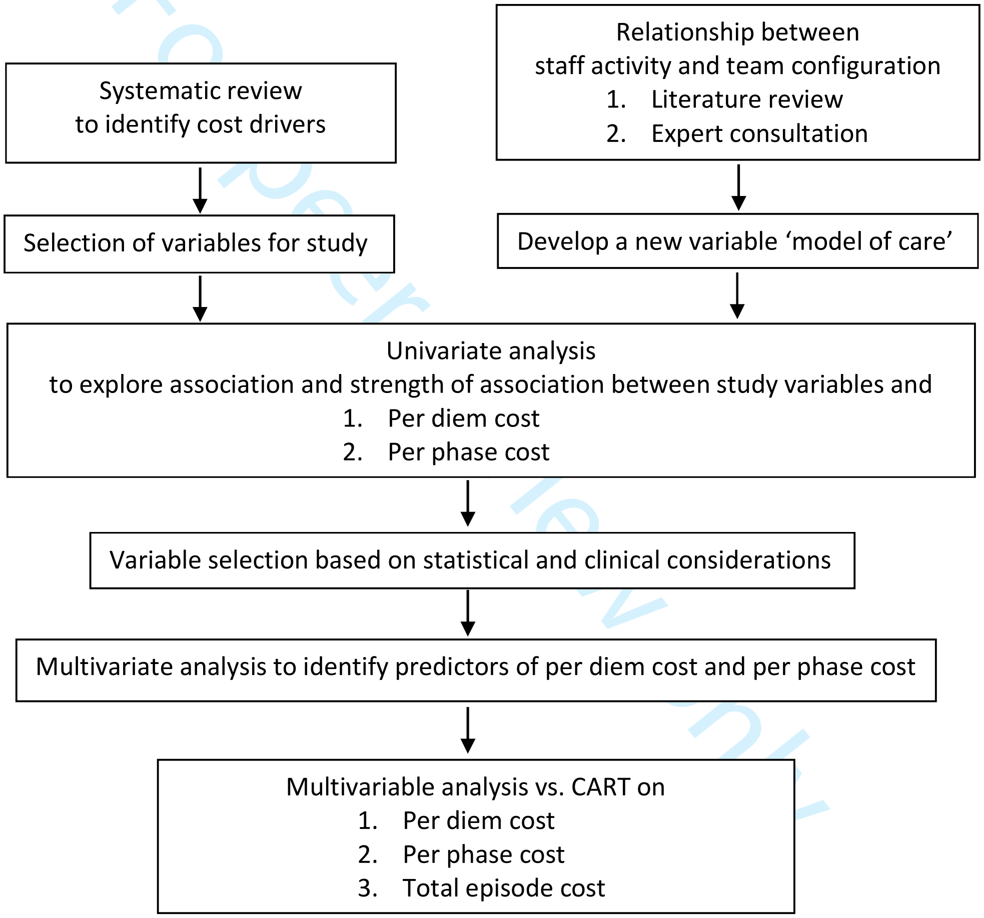

Figure 1 Data analysis flowchart

Qualitative analysis methods

Audio-recordings will be transcribed verbatim and checked for accuracy and entered into NVivo 10. Data will be independently coded and analysed by two members of the research team. We will adopt a similar approach to Pinnock,[48] undertaking a thematic and narrative analysis of interviews,

For peer review only - http://bmjopen.bmj.com/site/about/guidelines.xhtml

exploring how perspectives evolve over time, with detailed attention to patient and family perspectives on experience of care in each setting and transitions, including potential interventions (and hence cost levers or other triggers) to influence changes in settings of care.

Model development

The distribution of casemix criteria (cost predictors) in the participant population will be described based on phase of illness, functional status and problem severity, using parametric or non-parametric statistics, as appropriate. In order to reduce the number of cost predictors, we will initially use univariate analysis to explore the association and strength of association between casemix criteria and cost of the episode of care (quantifying cost by per diem cost, per phase cost and total cost of episode). We will use generalised linear model (multivariate regression model) to select variables that will then be applied in a hierarchical manner to form a branching classification in which each cost driver is incorporated only once.

Variables shown in Table 2 and 3 and the newly created categorical variable representing 'model of care' will be initially entered into the models and then non-significant predictors removed at each iteration using a Bootstrap sub-sampling strategy. Internal validation will be conducted by using such methods as bootstrapping or cross-validation to quantify any optimism in the predictive performance (calibration and discrimination) of the developed classification.

Model specification

The casemix classification for each outcome across three settings will be presented including all regression coefficients and model intercept (Figure 2). How to use the classification will be explained. Understanding how casemix variables predict cost of the episode of care enables a robust casemix classification to be developed. But in order for casemix adjustment to occur, there also needs to be a good understanding of how the casemix variables impact on clinical outcomes, as well as resource use. This casemix adjustment methodology is described in the recently published Department of Health document,[49] which recommends that significant casemix variables are identified, and that the size of their relationship with clinical outcomes is determined in advance.

Multi-level modelling will be undertaken to ensure both of these steps to be delivered in this study by determining (1) which casemix criteria are most strongly associated with clinical outcomes, allowing for clustering at the level of 'participant' (where episodes occur in the same individual), at the level of 'model of care' (where care is received according to similar models), and at the level of

For peer review only - http://bmjopen.bmj.com/site/about/guidelines.xhtml

'site' (where care is delivered in a similar way within site), and (2) which casemix criteria are the strongest cost predictor (including per diem cost, per phase cost, and total episode cost).

Model performance

The performance of the casemix classification will be assessed in both the development and validation datasets. Classification measures including sensitivity, specificity, predictive values, and net reclassification improvement will be reported and cut points selected a-priori. We will primarily focus on discrimination for the classification development phase, and assess both discrimination and calibration for the validation phase.[50] Discrimination of the classification will be measured using the concordance statistic and confidence intervals (c-statistic).[51] Calibration of the classification will be measured using calibration plot and Hosmer-Lemeshow goodness-of-fit test.[52]

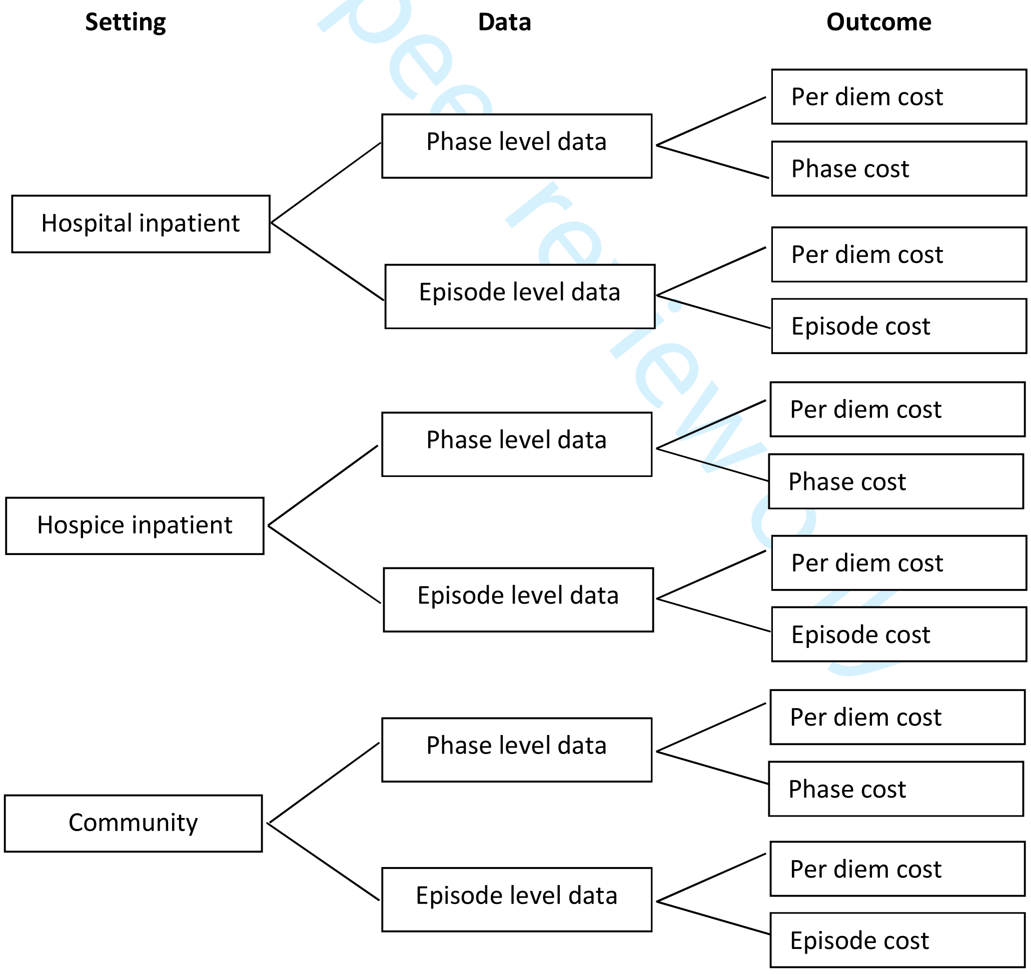

Figure 2 Casemix classification specification

For peer review only - http://bmjopen.bmj.com/site/about/guidelines.xhtml

Development vs. validation

Data will be collected from patients, caregivers, and clinicians during the validation phase to compare patient-reported problem severity i.e. 'felt' need collected in validation phase only (as measured by the patient-reported IPOS) and professional-reported problem severity i.e. 'normative' need (as measured by the clinician-reported IPOS, phase of illness, AKPS), by reporting correlation across levels of complexity and across conditions. We will assess how professional-reported measures relate to patient-reported outcomes across different levels of complexity (i.e. in relation to casemix classes), in order to determine optimal outcome measures and quality indicators.

We will compare data on resource use from two sources: i) clinician-completed brief PRUS, and ii) patients and family-completed Client Service Receipt Inventory, to better understand how the PRUS maps to patients' receipt of resources, and evaluate which generic services are utilised across different providers, regions, and the context of different models of specialist palliative care.

Ethical considerations

The study protocol and documents (e.g., the participant information sheet, consent and declaration form) have been approved by the NHS Health Research Authority London – Camberwell St Giles Research Ethics committee (15/LO/0887) for the development phase, and NHS Health Research Authority London – Bromley Research Ethics committee (16/LO/1021) for the validation phase.

All eligible participants are fully informed before consent is sought through the information sheets and verbal explanation on the aims and methods of the study and procedures that might be involved. Participants can withdraw at any time up to analysis of their data, without giving any reason. It is possible that participants may become distressed or raise issues during this study which raise concerns or warrant a change in their medical management, but we do not expect the questionnaires will themselves cause distress so much as uncover pre-existing distress which has not been acknowledged or recognised. Should this be the case, then our distress protocol will be followed. We anticipate distress will be infrequent, given the general nature of the questionnaires. It is likely that any distress will reflect advanced disease and experiences of care, and not the questionnaires themselves. All of the study team members have completed Good Clinical Practice training, and specific training on addressing distress in palliative care.

Our existing Patient/Public Advisory Group and extended Consumer Panel have been and will continue to be consulted throughout the study to ensure that the study is carried out in an ethical

For peer review only - http://bmjopen.bmj.com/site/about/guidelines.xhtml

and respectful way, and has the highest possible relevance and benefit to patients and families. A Project Steering Committee meets six monthly to monitor recruitment, review the detailed progress of the study, and make recommendations for overall direction and strategy.

Dissemination

This study will lead to patient benefit through improved matching of resources to needs at individual patient-level and will better enable the NHS to deliver high quality, patient-centred palliative care in last year of life. The results of the study will be published in peer-reviewed publications and will also be presented at national and international conferences.

For peer review only

For peer review only - http://bmjopen.bmj.com/site/about/guidelines.xhtml

Acknowledgements

This article presents independent research C-CHANGE funded by the National Institute for Health Research (NIHR) under the Programme Grants for Applied Research scheme (RP-PG-1210-12015) and the NIHR CLAHRC funding scheme, through the Collaboration for Leadership in Applied Health Research and Care (CLAHRC) in South London. CLAHRC South London is part of the NIHR and is a partnership between King's Health Partners, St George's, University of London, and St George's Healthcare NHS Trust. The C-CHANGE research team acknowledges the support of the National Institute of Health Research Clinical Research Network (NIHR CRN).

Contributors

PG drafted the study protocol with input from all authors. MD, JMD and AD provided statistical advice and support. AMF, SMO, CP, SP and PG contributed to the acquisition and interpretation of data for the work. IJH and FEM conceived the study and were in charge of overall direction and planning. KE provided experience from the Australian palliative care casemix development.[13] FEM is the Chief Investigator and designed the C-CHANGE research programme of which this is part. All authors provided critical feedback, helped revise the manuscript, and are accountable for the accuracy and integrity of all aspects of the work.

Funding

This work was supported by NIHR Programme Grants for Applied Research (Grant number RP-PG-1210-12015).

Disclaimer

The views and opinions expressed are those of the authors and do not necessarily reflect those of the NHS, the NIHR, MRC, CCF, NETSCC, the NIHR Programme Grants for Applied Research, or the Department of Health.

Competing interests

None declared.

Ethics approval

The NHS Health Research Authority (HRA) Approval.

For peer review only

For peer review only - http://bmjopen.bmj.com/site/about/guidelines.xhtml

References

1. Currow DC, Abernethy AP and Fazekas BS. Specialist palliative care needs of whole populations: a feasibility study using a novel approach. *Palliative Medicine*. 2004; 18: 239-47.
2. Fitch MI. Needs of patients living with advanced disease. *Canadian Oncology Nursing Journal* 2005; 15: 230-42.
3. Department of Health. *Assessing Health Care Needs.* London: National Health Service Management Executive, 1991.
4. The Economist Intelligence Unit. *The 2015 Quality of Death Index: Ranking Palliaitve Care across the World.* 2015.
5. Sleeman KE, Davies JM, Verne J, Gao W and Higginson IJ. The changing demographics of inpatient hospice death: Population-based cross-sectional study in England, 1993–2012. *Palliative Medicine*. 2016; 30: 45-53.
6. Rosenwax L, Spilsbury K, McNamara BA and Semmens JB. A retrospective population based cohort study of access to specialist palliative care in the last year of life: who is still missing out a decade on? *BMC Palliative Care*. 2016; 15: 46.
7. Hughes-Hallett T, Craft A, Davies C, Mackay I and Nielsson T. Funding the right care and support for everyone: Creating a fair and transparent funding system; the final report of the palliative care funding review. *Palliative Care Funding Review*. 2011.
8. Lancaster H, Finlay I, Downman M and Dumas J. Commissioning of specialist palliative care services in England. *BMJ Supportive & Palliative Care*. 2017. DOI:10.1136/bmjspcare-2016-001119.
9. Etkind SN, Bone AE, Gomes B, et al. How many people will need palliative care in 2040? Past trends, future projections and implications for services. *BMC Medicine*. 2017; 15: 102.
10. Lee LA, Eagar KM and Smith MC. Subacute and non-acute casemix in Australia. *The Medical Journal of Australia*. 1998; 169: 22-5.
11. Eagar K, Cromwell D, Kennedy C and Lee L. Classifying sub-acute and non-acute patients: Results of the New South Wales Casemix Area Network study. *Australian Health Review*. 1997; 20: 26-42.
12. Turner-Stokes L, Sutch S, Dredge R and Eagar K. International casemix and funding models: lessons for rehabilitation. *Clinical rehabilitation*. 2011; 26: 195-208.
13. Eagar K, Gordon R, Hodkinson A, et al. *The Australian National Sub-Acute and Non-Acute Patient Classification (AN-SNAP): report of the National Sub-Acute and Non-Acute Casemix Classification Study.* Centre for Health Service Development, University of Wollongong1997.
14. Eagar K, Green J and Gordon R. An Australian casemix classification for palliative care: technical development and results. *Palliative Medicine*. 2004; 18: 217-26.
15. Eagar K, Watters P, Currow DC, Aoun SM and Yates P. The Australian Palliative Care Outcomes Collaboration (PCOC)--measuring the quality and outcomes of palliative care on a routine basis. *Australian Health Review*. 2010; 34: 186-92.
16. Eagar K, Gordon R, Green J and Smith M. An Australian casemix classification for palliative care: lessons and policy implications of a national study. *Palliative Medicine*. 2004; 18: 227-33.
17. Collins GS, Reitsma JB, Altman DG and Moons KM. Transparent reporting of a multivariable prediction model for individual prognosis or diagnosis (tripod): The tripod statement. *Annals of Internal Medicine*. 2015; 162: 55-63.
18. Hinds J. *National Survey of Patient Activity Data for Specialist Palliative Care Services: Minimum Data Set (MDS) Full Report for the Year 2014-2015*. London: The National Council for Palliative Care, 2016.
19. Castro FG, Kellison JG, Boyd SJ and Kopak A. A methodology for conducting integrative mixed methods research and data analyses. *Journal of Mixed Methods Research*. 2010; 4: 342-60.
20. Hjermstad M, Loge JH and Kaasa S. Methods for assessment of cognitive failure and delirium in palliative care patients: implications for practice and research. *Palliative Medicine*. 2004; 18: 494-506.

For peer review only - http://bmjopen.bmj.com/site/about/guidelines.xhtml

21. Radbruch L, Sabatowski R, Loick G, et al. Cognitive impairment and its influence on pain and symptom assessment in a palliative care unit: development of a Minimal Documentation System. *Palliative Medicine*. 2000; 14: 266-76.
22. Higginson IJ, McCrone P, Hart SR, Burman R, Silber E and Edmonds PM. Is Short-Term Palliative Care Cost-Effective in Multiple Sclerosis? A Randomized Phase II Trial. *Journal of Pain and Symptom Management*. 2009; 38: 816-26.
23. Bausewein C, Booth S, Gysels M, Kühnbach R, Haberland B and Higginson IJ. Individual breathlessness trajectories do not match summary trajectories in advanced cancer and chronic obstructive pulmonary disease: results from a longitudinal study. *Palliative Medicine*. 2010; 24: 777-86.
24. Buller T. Advance consent, critical interests and dementia research. *Journal of Medical Ethics*. 2015; 41: 701-7.
25. Mather H, Guo P, Firth A, et al. Phase of Illness in palliative care: Cross-sectional analysis of clinical data from community, hospital and hospice patients. *Palliative Medicine*. DOI: 10.1177/0269216317727157.
26. Masso M, Allingham SF, Banfield M, et al. Palliative Care Phase: Inter-rater reliability and acceptability in a national study. *Palliative Medicine*. 2015; 29: 22-30.
27. Coventry PA, Grande GE, Richards DA and Todd CJ. Prediction of appropriate timing of palliative care for older adults with non-malignant life-threatening disease: a systematic review. *Age and Ageing*. 2005; 34: 218-27.
28. Franks PJ, Salisbury C, Bosanquet N, et al. The level of need for palliative care: a systematic review of the literature. *Palliative Medicine*. 2000; 14: 93-104.
29. Luddington L, Cox S, Higginson I and Livesley B. The need for palliative care for patients with non-cancer diseases: a review of the evidence. *International Journal of Palliative Nursing*. 2001; 7: 221-6.
30. Tebbit P. *Population-based Needs Assessment for Palliaitve and End of Life Care*. London: National Council for Palliative Care, 2008.
31. Drummond M, Weatherly H and Ferguson B. *Economic Evaluation of Health Interventions*. 2008.
32. Curtis L. *Unit Costs of Health and Social Care 2014*. Canterbury: Personal Social Services Research Unit, University of Kent, 2014.
33. *Joint Formulary Committe. British National Formulary (BNF) 68*. London: BMJ Publishing Group Ltd and Royal Pharmaceutical Society, 2014.
34. Department of Health. *NHS reference costs 2013 to 2014*. 2014.
35. Wolff J, McCrone P, Patel A, Auber G and Reinhard T. A time study of physicians' work in a German University Eye Hospital to estimate unit costs. *PLOS ONE*. 2015; 10: e0121910.
36. Sutherland JM. Pricing hospital care: Global budgets and marginal pricing strategies. *Health Policy*. 2015.
37. Tan SS, Bakker J, Hoogendoorn ME, et al. Direct cost analysis of Intensive Care Unit stay in four European countries: Applying a standardized costing methodology. *Value in Health*. 2012; 15: 81-6.
38. Department of Health. NHS costing manual. 2012.
39. King J, Gibbons E, Graham C and Walsh J. Developing measures of people's self-reported experiences of integrated care. *Picker Institute Europe and University of Oxford*. 2013.
40. Koffman J, Higginson IJ, Hall S, Riley J, McCrone P and Gomes B. Bereaved relatives' views about participating in cancer research. *Palliative Medicine*. 2012; 26: 379-83.
41. Addington-Hall J and McPherson C. After-death interviews with surrogates/bereaved family members: Some issues of validity. *Journal of Pain and Symptom Management*. 2001; 22: 784-90.
42. Costantini M, Beccaro M and Merlo F. The last three months of life of Italian cancer patients. Methods, sample characteristics and response rate of the Italian Survey of the Dying of Cancer (ISDOC). *Palliative Medicine*. 2005; 19: 628-38.

For peer review only - http://bmjopen.bmj.com/site/about/guidelines.xhtml

43. VanVoorhis CW and Morgan BL. Understanding power and rules of thumb for determining sample sizes. *Tutorials in Quantitative Methods for Psychology*. 2007; 3: 43-50.
44. Ogundimu EO, Altman DG and Collins GS. Adequate sample size for developing prediction models is not simply related to events per variable. *Journal of Clinical Epidemiology*. 2016; 76: 175-82.
45. Currow DC, Allingham S, Yates P, Johnson C, Clark K and Eagar K. Improving national hospice/palliative care service symptom outcomes systematically through point-of-care data collection, structured feedback and benchmarking. *Supportive Care in Cancer*. 2015; 23: 307-15.
46. Gao J, Johnston GM, Lavergne MR and McIntyre P. Identifying population groups with low palliative care program enrolment using classification and regression tree analysis. *Journal of Palliative Care*. 2011; 27: 98-106.
47. Lemon SC, Roy J, Clark MA, Friedmann PD and Rakowski W. Classification and regression tree analysis in public health: Methodological review and comparison with logistic regression. *Annals of Behavioral Medicine*. 2003; 26: 172-81.
48. Pinnock H, Kendall M, Murray SA, et al. Living and dying with severe chronic obstructive pulmonary disease: multi-perspective longitudinal qualitative study. *BMJ*. 2011; 342.
49. Department of Health. *Patient Reported Outcome Measures (PROMs) in England: the Case-mix Adjustment Methodology.* 2012.
50. Steyerberg EW, Vickers AJ, Cook NR, et al. Assessing the performance of prediction models: a framework for some traditional and novel measures. *Epidemiology (Cambridge, Mass)*. 2010; 21: 128-38.
51. Cook NR. Statistical evaluation of prognostic versus diagnostic models: Beyond the ROC curve. *Clinical Chemistry*. 2007; 54: 17.
52. Austin PC and Steyerberg EW. Graphical assessment of internal and external calibration of logistic regression models by using loess smoothers. *Statistics in Medicine*. 2014; 33: 517-35.

For peer review only

For peer review only - http://bmjopen.bmj.com/site/about/guidelines.xhtml

TRIPOD Checklist: Prediction Model Development and Validation

Section/Topic	Item		Checklist Item	Page
**Title and abstract**				
Title	1	D;V	Identify the study as developing and/or validating a multivariable prediction model, the target population, and the outcome to be predicted.	1
Abstract	2	D;V	Provide a summary of objectives, study design, setting, participants, sample size, predictors, outcome, statistical analysis, results, and conclusions.	1
**Introduction**				
Background and objectives	3a	D;V	Explain the medical context (including whether diagnostic or prognostic) and rationale for developing or validating the multivariable prediction model, including references to existing models.	3-4
	3b	D;V	Specify the objectives, including whether the study describes the development or validation of the model or both.	3-4
**Methods**				
Source of data	4a	D;V	Describe the study design or source of data (e.g., randomized trial, cohort, or registry data), separately for the development and validation data sets, if applicable.	4-5
	4b	D;V	Specify the key study dates, including start of accrual; end of accrual; and, if applicable, end of follow-up.	5-6
Participants	5a	D;V	Specify key elements of the study setting (e.g., primary care, secondary care, general population) including number and location of centres.	5-6
	5b	D;V	Describe eligibility criteria for participants.	5-6
	5c	D;V	Give details of treatments received, if relevant.	5-6
Outcome	6a	D;V	Clearly define the outcome that is predicted by the prediction model, including how and when assessed.	6-7
	6b	D;V	Report any actions to blind assessment of the outcome to be predicted.	N/A
Predictors	7a	D;V	Clearly define all predictors used in developing the multivariable prediction model, including how and when they were measured.	7-9
	7b	D;V	Report any actions to blind assessment of predictors for the outcome and other predictors.	N/A
Sample size	8	D;V	Explain how the study size was arrived at.	9-10
Missing data	9	D;V	Describe how missing data were handled (e.g., complete-case analysis, single imputation, multiple imputation) with details of any imputation method.	10
Statistical analysis methods	10a	D	Describe how predictors were handled in the analyses.	10-12
	10b	D	Specify type of model, all model-building procedures (including any predictor selection), and method for internal validation.	12-13
	10c	V	For validation, describe how the predictions were calculated.	12-13
	10d	D;V	Specify all measures used to assess model performance and, if relevant, to compare multiple models.	12-13
	10e	V	Describe any model updating (e.g., recalibration) arising from the validation, if done.	12-13
Risk groups	11	D;V	Provide details on how risk groups were created, if done.	12-13
Development vs. validation	12	V	For validation, identify any differences from the development data in setting, eligibility criteria, outcome, and predictors.	14
**Results**				
Participants	13a	D;V	Describe the flow of participants through the study, including the number of participants with and without the outcome and, if applicable, a summary of the follow-up time. A diagram may be helpful.	N/A
	13b	D;V	Describe the characteristics of the participants (basic demographics, clinical features, available predictors), including the number of participants with missing data for predictors and outcome.	N/A
	13c	V	For validation, show a comparison with the development data of the distribution of important variables (demographics, predictors and outcome).	N/A
Model development	14a	D	Specify the number of participants and outcome events in each analysis.	N/A
	14b	D	If done, report the unadjusted association between each candidate predictor and outcome.	N/A
Model specification	15a	D	Present the full prediction model to allow predictions for individuals (i.e., all regression coefficients, and model intercept or baseline survival at a given time point).	N/A
	15b	D	Explain how to use the prediction model.	N/A
Model performance	16	D;V	Report performance measures (with CIs) for the prediction model.	N/A
Model-updating	17	V	If done, report the results from any model updating (i.e., model specification, model performance).	N/A
**Discussion**				
Limitations	18	D;V	Discuss any limitations of the study (such as nonrepresentative sample, few events per predictor, missing data).	14-15
Interpretation	19a	V	For validation, discuss the results with reference to performance in the development data, and any other validation data.	N/A
	19b	D;V	Give an overall interpretation of the results, considering objectives, limitations, results from similar studies, and other relevant evidence.	N/A
Implications	20	D;V	Discuss the potential clinical use of the model and implications for future research.	14-15
**Other information**				
Supplementary information	21	D;V	Provide information about the availability of supplementary resources, such as study protocol, Web calculator, and data sets.	N/A
Funding	22	D;V	Give the source of funding and the role of the funders for the present study.	16

*Items relevant only to the development of a prediction model are denoted by D, items relating solely to a validation of a prediction model are denoted by V, and items relating to both are denoted D;V. We recommend using the TRIPOD Checklist in conjunction with the TRIPOD Explanation and Elaboration document.

BMJ Open

Development and validation of a casemix classification to predict costs of specialist palliative care provision across inpatient hospice, hospital, and community settings in the UK: A study protocol

Journal:	*BMJ Open*
Manuscript ID	bmjopen-2017-020071.R1
Article Type:	Protocol
Date Submitted by the Author:	02-Jan-2018
Complete List of Authors:	Guo, Ping; King's College London, Cicely Saunders Institute, Department of Palliative Care, Policy and Rehabilitation<br>Dzingina, Mendwas; King's College London, Cicely Saunders Institute, Department of Palliative Care, Policy and Rehabilitation<br>Firth, Alice; King's College London, Cicely Saunders Institute, Department of Palliative Care, Policy and Rehabilitation<br>Davies, Joanna; King's College London, Cicely Saunders Institute, Department of Palliative Care, Policy and Rehabilitation<br>Douiri, Abdel; King's College London, Primary Care And Public Health Sciences<br>O'Brien, Suzanne; King's College London, Cicely Saunders Institute, Department of Palliative Care, Policy and Rehabilitation<br>Pinto, Cathryn; King's College London, Cicely Saunders Institute, Department of Palliative Care, Policy and Rehabilitation<br>Pask, Sophie; King's College London, Cicely Saunders Institute, Department of Palliative Care, Policy and Rehabilitation<br>Higginson, Irene; Kings College London, Cicely Saunders Institute, Department of Palliative Care, Policy and Rehabilitation<br>Eagar, Kathy; University of Wollongong, Australian Health Services Research Institute, Centre for Health Service Development<br>Murtagh, Fliss; University of Hull, Wolfson Palliative Care Research Centre, Hull York Medical School
**Primary Subject Heading**:	Palliative care
Secondary Subject Heading:	Health services research
Keywords:	Adult palliative care < PALLIATIVE CARE, Casemix classification, Validation, Cost predictors

SCHOLARONE™
Manuscripts

TITLE PAGE

Full title:

Development and validation of a casemix classification to predict costs of specialist palliative care provision across inpatient hospice, hospital, and community settings in the UK: A study protocol

Author details:

Dr Ping Guo,
Project Manager and Research Associate
Cicely Saunders Institute, Department of Palliative Care, Policy & Rehabilitation, King's College London, Bessemer Road, SE5 9PJ, UK
Correspondence to P. Guo: Email: ping.guo@kcl.ac.uk Telephone: 020 7848 5523

Dr Mendwas Dzingina, Cicely Saunders Institute, Department of Palliative Care, Policy & Rehabilitation, King's College London, Bessemer Road, SE5 9PJ, UK

Alice M Firth, Cicely Saunders Institute, Department of Palliative Care, Policy & Rehabilitation, King's College London, Bessemer Road, SE5 9PJ, UK

Joanna M Davies, Cicely Saunders Institute, Department of Palliative Care, Policy & Rehabilitation, King's College London, Bessemer Road, SE5 9PJ, UK

Dr Abdel Douiri, Department of Primary Care and Public Health Sciences, Addison House, Guy's Campus, King's College London, UK

Suzanne M O'Brien, Cicely Saunders Institute, Department of Palliative Care, Policy & Rehabilitation, King's College London, Bessemer Road, SE5 9PJ, UK

Cathryn Pinto, Cicely Saunders Institute, Department of Palliative Care, Policy & Rehabilitation, King's College London, Bessemer Road, SE5 9PJ, UK

Sophie Pask, Cicely Saunders Institute, Department of Palliative Care, Policy & Rehabilitation, King's College London, Bessemer Road, SE5 9PJ, UK

Professor Irene J Higginson, Cicely Saunders Institute, Department of Palliative Care, Policy & Rehabilitation, King's College London, Bessemer Road, SE5 9PJ, UK

Professor Kathy Eagar, University of Wollongong, Australian health Services Research Institute, Centre for Health Service Development, Wollongong, Australia

Professor Fliss EM Murtagh, Wolfson Palliative Care Research Centre, Hull York Medical School, University of Hull, UK

For peer review only - http://bmjopen.bmj.com/site/about/guidelines.xhtml

Development and validation of a casemix classification to predict costs of specialist palliative care provision across inpatient hospice, hospital, and community settings in the UK: A study protocol

ABSTRACT

Introduction: Provision of palliative care is inequitable with wide variations across conditions and settings in the UK. Lack of a standard way to classify by case complexity is one of the principle obstacles to addressing this. We aim to develop and validate a casemix classification to support the prediction of costs of specialist palliative care provision.

Methods and analysis:

Phase I - A cohort study to determine the variables and potential classes to be included in a casemix classification. Data are collected from clinicians in palliative care services across inpatient hospice, hospital, and community settings on: patient demographics, potential complexity/casemix criteria, and patient-level resource use. Cost predictors are derived using multivariate regression and then incorporated into a classification using Classification and Regression Trees (CART). Internal validation will be conducted by bootstrapping to quantify any optimism in the predictive performance (calibration and discrimination) of the developed classification.

Phase II - A mixed methods cohort study across settings for external validation of the classification developed in Phase I. Patient and family caregiver data will be collected longitudinally on demographics, potential complexity/casemix criteria, and patient-level resource use. This will be triangulated with data collected from clinicians on potential complexity/casemix criteria and patient-level resource use, and with qualitative interviews with patients and caregivers about care provision across difference settings. The classification will be refined on the basis of its performance in the validation data set.

Ethics and dissemination: The study has been approved by the National Health Service (NHS) Health Research Authority Research Ethics Committee. The results are expected to be disseminated in 2018 through papers for publication in major palliative care journals; policy briefs for clinicians, commissioning leads, and policy makers; and lay summaries for patients and public.

Study registration number: ISRCTN90752212

For peer review only - http://bmjopen.bmj.com/site/about/guidelines.xhtml

Strengths and limitations of this study

- This is the first study in the UK to determine the variables and potential classes to be included in a casemix classification, which predicts costs of specialist palliative care provision across inpatient hospice, hospital, and community settings.
- Transparent reporting of a multivariable prediction model for individual prognosis or diagnosis (TRIPOD) is used to guide the reporting of this study.
- The newly developed casemix classification in phase I will be externally validated by using a different dataset in phase II.
- This study also promotes the implementation of outcome measures in palliative care into routine clinical practice across different settings of care.
- This is a UK focused study so the casemix classification may not be directly applied to palliative care in other countries without further investigation and refinement.

For peer review only

For peer review only - http://bmjopen.bmj.com/site/about/guidelines.xhtml

INTRODUCTION

People with advanced and incurable illness often suffer complex and multiple symptoms and psychosocial concerns because of their illness or impending death.[1, 2] Their families may provide day-to-day care, as well as being affected by their own anxieties, concerns, and potential losses. These bring increased need for health and social care, with need being defined as 'the ability to benefit from health or social care interventions'.[3] Palliative care has developed to meet the needs of these patients and families, which addresses physical/psychological symptoms and provides social, practical and spiritual support. The UK ranks first in the 2015 Quality of Death Index - a measure of the quality of dying in 80 countries,[4] and the hospice movement in the UK has provided a model of good palliative care for those in need.

However, marked inequities exist in provision of palliative care across England. Older people and those with non-cancer diagnoses are less likely to access specialist palliative care.[5, 6] There are also major geographical variations, ranging from £186 to £6,213 per person across different Primary Care Trusts in 2010,[7, 8] often resulting in a poor match between individual needs, resources provided to meet those needs, and health outcomes achieved. With an ageing population and increasing rates of chronic diseases, the growing healthcare burden is overwhelmingly challenging in terms of health resource allocation around the UK.[9] With recognition of the constraints on resources, there is support for the systematic approach to mapping individual needs accurately and improving the quality and efficiency of palliative care. This has been endorsed as a high priority nationally.[7]

The Diagnosis Related Group is a useful classification of healthcare needs driven by the diagnosis but is inappropriate for palliative care, because palliative care needs are not driven by the diagnosis but by factors such as functional status and symptoms. Palliative care needs a consistent method of classifying types of patients with complexity of needs, treatment, and costs, using casemix criteria.[10-12] It is necessary to identify those with more complex palliative needs, requiring more resources. An Australian casemix classification for palliative care was developed in 1997, empirically tested and progressively refined over time.[13-15] The Australian casemix classification consists of classes defined by five criteria including Phase of Illness, problem severity, functional status and dependency, age, model of care, as most strongly predictive of resource use.[16] Full class definition and categorisation is available at http://www.pcoc.org.au/. Its implementation proved the possibility of consistently and routinely collecting data in practice in Australia.[16] However, due to variations in outcome measures collected and palliative care provided between countries, it is unclear whether any existing palliative care classification can be easily applied to the UK to address unmet needs and resolve the inequity.

For peer review only - http://bmjopen.bmj.com/site/about/guidelines.xhtml

This study is part of a five year NIHR funded C-CHANGE programme (RP-PG-1210-12015). It aims to develop and validate a casemix classification for palliative care in the UK. Specific objectives are:

- To determine the cost predictors of specialist palliative care, adjusting for important confounding factors including unmet needs.
- To develop and validate a casemix classification for specialist palliative care.

METHODS AND ANALYSIS

The Transparent Reporting of a multivariable prediction model for Individual Prognosis Or Diagnosis (TRIPOD) Statement [17] is used to guide the reporting of this study protocol.

Study design and source of data

This study consists of two phases: Development phase (Phase I) and validation phase (Phase II). In phase I, we will identify variables that predict costs to be included in casemix classification through a cohort study with only clinician data, and develop potential casemix classes for palliative care which will be tested in individual episodes of care (internal validation). In phase II, we will prospectively validate these potential classes in a new cohort study with patient, caregiver, and clinician data, and include qualitative interviews to ensure it works during care transitions and longitudinally over the course of illness (external validation).

Phase I – Development phase

This is a cohort study to determine the variables and potential classes to be included in a casemix classification, based on individual episodes of care across inpatient (hospice and hospital) and community settings. An 'episode of care' starts when a patient is admitted to inpatient services or begins to receive specialist palliative care from a community-based or outpatient service, and ends when a patient is discharged from that service or dies. The median duration of an episode of care is expected to be under 14 days in inpatient and at a median of 72 days in community settings.[18]

Phase I was conducted between 31st July 2015 and 30th September 2016 and follow-up ended 30th November 2016. Data were collected from clinicians through surveys, including demographic/clinical data, episode start/end data, patient attributes that predict palliative care resource consumption (e.g., Phase of Illness, functional status, and problem severity as identified in the Australian casemix classification study [13, 14, 16]), plus information on patient-level resource use (staff activity and clinical services) in specialist palliative care settings.

For peer review only - http://bmjopen.bmj.com/site/about/guidelines.xhtml

For peer review only

Phase II – Validation phase

This phase is a mixed methods cohort study with a concurrent nested design:[19]

- Quantitative main component - Prospectively collect data from patients, caregivers and clinicians on palliative care needs, concerns, outcomes, and resources use.
- Qualitative nested component - Longitudinal interviews with a subsample of participants to understand care provision in each setting and transitions between settings.

Phase II will be conducted between 1st November 2016 and 31st December 2017, with follow-up ending on 28th February 2018. The same variables and measurements as Phase I will be collected from clinicians. Additionally, patient/caregiver participants will provide data on symptoms/concerns, experience of care, and their use of services, and be followed through all episodes of care from recruitment to death or the end of this study. A post bereavement survey will be conducted with caregivers where appropriate to identify symptoms/concerns immediately prior to death and support needs after death.

Participants

Phase I – Development phase

All adults (≥18 years) receiving specialist palliative care newly admitted in 10 participating sites during the study period (2 sites providing hospital advisory services, 1 providing community-based service only, 6 providing hospice inpatient and community-based services, 1 providing hospital advisory and community-based services) were included, regardless of primary diagnosis. We selected these sites with the aim of ensuring a representative sample in terms of population demographics (age distribution, ethnicity, socio-economic status, and rural/urban composition). Written informed consent was taken by a qualified member of the research or clinical team. This phase included individuals with limited, fluctuating, diminishing or lack of capacity, vital to ensure that any casemix classification is applicable to all palliative patients, not just those able to consent. It is recognised that a high proportion of those have impaired capacity, and these patients may need palliative care and resources most.[20-23] Therefore, if the clinician assessed that the patient did not currently have the capacity to give consent, assent was sought from an accompanying family member, or failing that, a staff member to whom the patient was known. Where a formally appointed Power of Attorney existed, this took precedence.

Phase II – Validation phase

For peer review only - http://bmjopen.bmj.com/site/about/guidelines.xhtml

All adults (≥18 years) receiving specialist palliative care newly admitted from 14 sites during the study period (4 extra sites to increase recruitment) will be eligible, regardless of primary diagnosis. Written informed consent will be sought from eligible patients (and their family caregivers) at the outset, including advance consent for study follow-up if capacity is lost. This study is of minimal risk with participants not exposed to undue harm.[24]

For both Phase I and II, a list of study sites can be obtained via: https://www.kcl.ac.uk/nursing/departments/cicelysaunders/research/studies/c-change/c-change.aspx.

Since transition of care is important for casemix, patient participants who transfer between settings (and caregiver participants) will be followed up. We will verbally confirm continuing consent with each participant when making such contact. If capacity of patient participants is lost, we will seek advice from a personal consultee on whether the patient should remain in the study to verify no change of decisions has been expressed by the patient prior to loss of capacity. On recruitment, participants will be asked if willing to be interviewed at a later stage. To capture variation in age, gender, diagnosis, and geographical location, we will purposively select 20-25 patients and family caregivers with at least two transitions of care for face-to-face interviews. Each semi-structured interview will last 40 minutes, but will be guided by each participant. In order to provide information on how care transitions might be better negotiated to improve outcomes and experiences, the interviews will cover communication, coordination of care, information/support needs, discharge planning, and experience of transitions.

Outcome

The outcome is cost of specialist palliative care (including per diem cost, per phase cost, and total episode cost) captured by: (1) staff activity matrix in both the development and validation phase, (2) the Palliative care Resource Use Score (PRUS) in both phases, and (3) Palliative Client Services Receipt Inventory (Pall-CSRI) in validation phase only.

Staff activity

At every contact, nurses, doctors, and allied health professionals will record the time spent on face-to-face and phone contacts, and patient-level administrative time per shift using the staff activity matrix paper version (Table 1). Staff training and site feedback of activity data were conducted regularly to improve and optimise data quality.

For peer review only - http://bmjopen.bmj.com/site/about/guidelines.xhtml

For peer review only

Table 1 Staff activity matrix (Each box is completed in units of 5 minutes, from 0 up to 120 minutes)

Staff time (mins)	Patient	Family/carer	Professional (internal)	Professional (external)
Face-to-face/phone time				
Administrative time				

Palliative care Resource Use Score (PRUS)

PRUS will be collected by staff at change of 'Phase of Illness'[25, 26] and at the end of each episode by recording the following information for the phase which has ended:

- Level of professional input (e.g., registered nurses, specialist palliative nursing staff, palliative doctors, and social workers) and whether this met patient/family needs.
- Level of 'out of hours' services by professional designation.
- Equipment, high cost drugs, diagnostic tests, and medical imaging. Within PRUS, the member of staff is asked to indicate whether or not equipment, high cost oral/transdermal medications, injectable medications/interventions, and medical imaging or tests were received by the participant, from a list derived from prior work to identify these options.
- Any unmet needs which provide insight into any gaps between needs and provision.[27-29] When completing PRUS, the member of staff is asked to estimate if any patients' or families' unmet needs existed, and if so, what needs were unmet.

Palliative Client Services Receipt Inventory (Pall-CSRI)

The Pall-CSRI is a patient/caregiver completed inventory of palliative care services received, and adapted from those used with palliative care populations.[30] It takes approximately 20 minutes to complete and collects retrospective information about the use of health/social care services, medication, living situation, income, employment and benefits, plus informal care. The Pall-CSRI will be collected three monthly or at the end of each episode of care whichever is earlier in the validation phase.

In the development phase, only direct care costs will be calculated but not productivity losses.[31] Direct care cost refers to all costs due to resource use that are completely attributable to the use of a health care intervention or illness, which can be split into direct medical costs (the cost of a defined intervention and all follow-up costs for other medication and health care interventions in ambulatory, inpatient, nursing care, home, or other relevant settings) and direct non-medical costs

For peer review only - http://bmjopen.bmj.com/site/about/guidelines.xhtml

(e.g., transportation costs and additional paid caregiver time).[32] In the validation phase, we will use the same health services costing perspective as in the development phase, but informal care costs are also considered to be in-scope and will be analysed separately, hypothesising that informal care costs are (1) greater in those with unmet needs, and (2) greater in those with non-cancer conditions. The costs of each resource item will be calculated by combining the resource use data with appropriate unit cost obtained from recognised sources including the annual compendium produced by the Personal Social Services Research Unit (PSSRU),[33] prices on the NHS supply chain website http://www.supplychain.nhs.uk, British national formulary,[34] and NHS reference costs.[35] The focus will be on staff time - the main resource in palliative care documented in a variety of settings and countries.[36-38] We will attribute costs according to a standard costing methodology adopted from the current NHS costing principles.[39]

Predictors (casemix criteria)

Proposed cost predictors can be categorised into three groups: (1) predictors collected from clinicians, (2) predictors collected from patients/family caregivers, and (3) model of care.

Predictors collected from clinicians

In both the development and validation phases, clinicians will record demographic, clinical and episode start administrative data. Episode end data will be collected at the end of each episode of care. Casemix data include Phase of Illness (stable, unstable, deteriorating, dying), functional status (measured by Australia-modified Karnofsky Performance Status - AKPS), dependency (Modified Barthel Index), problem severity (Integrated Palliative care Outcome Scale - IPOS staff version), and caregiver needs (Table 2). The measures are available direct from: Phase of Illness (www.pcoc.org.au), AKPS,[40] Modified Barthel Index,[41] and IPOS (www.pos-pal.org) respectively. Phase of Illness will be assessed daily for people receiving inpatient care and at each face-to-face contact basis for those receiving community-based care. Other variables including AKPS, Modified Barthel Index, IPOS-staff (and caregiver needs when feasible) will be collected at the start of episode, end of episode, and at change of Phase of Illness.

Table 2 Data from clinicians in both development phase and validation phase

**Type of data**	**Proposed cost predictors**
Demographic data	Age
	Gender
	Postcode

For peer review only - http://bmjopen.bmj.com/site/about/guidelines.xhtml

Type of data	Proposed cost predictors
	Ethnicity
	Marital status
	Living circumstances
	Need for interpreter
	Setting of care
Clinical data	Primary diagnosis
	Secondary diagnoses
	Co-morbidities
Episode start and end data	Episode start date
	Episode end date
	Endpoint of episode (discharged or died).
	Discharge destination, if discharged
Key casemix	Phase of Illness at start of episode
	Functional status (AKPS)
	Dependency (Modified Barthel Index)
	Problem severity (IPOS – staff version)
	Family/caregiver needs

Predictors collected from patients and family caregivers

Variables (Table 3) will be collected in the validation phase from patients/caregivers using face-to-face/telephone contacts and postal questionnaires according to their preferences. IPOS - patient version and Distress Thermometer will be collected from patients at the start of episode, at change of Phase of Illness, and at the end of episode. Views on Care (VoC) and SF-12v2 will be collected at the start and end of each episode of care. Selected questions capturing experiences of integrated care[42] will be collected at the end of episode only.

All caregiver reported measures will be collected at the start of episode, at change of Phase of Illness, and at the end of episode. Where phase length exceeds 4 weeks, data collected from patients (and caregivers) will be captured as a change of phase. If the patient is transferred to an out of scope inpatient setting, we will follow up with the patient when he or she gets home and retrospectively collect minimum information about this episode of care.

For peer review only - http://bmjopen.bmj.com/site/about/guidelines.xhtml

Table 3 Predictors collected from patients and family caregivers in validation phase only

**Data collection from patients**	**Data collection from family caregivers**
IPOS – patient version	Basic demographic information
Distress Thermometer	Distress Thermometer
Views on Care (VoC)	2 caregiver questions
SF-12v2*	Zarit 6 items
Patient experiences of integrated care	

* The measure could be completed by a family caregiver if the patient is too unwell to complete.

If the patient is deceased, data such as SF-12v2 and Pall-CSRI will be collected from the participating caregiver three months after death by postal questionnaire, along with the bereavement support information and contacts. The time between death and the postal questionnaire may influence caregivers' willingness to share their views; shorter periods possibly being too upsetting to contemplate involvement.[43] There is mixed evidence of the best time to make contact with potential participants for follow-back surveys[44, 45] and 3-4 months post bereavement is considered acceptable by bereaved families.[30]

Model of care

Models of care may be a stronger cost driver than patient-level variables.[13, 14, 16] Specialist palliative care services have various configurations of staff, interventions, and other characteristics. Currently, there is no consistent way to define models of palliative care. A separate, parallel study will be conducted in which service-level data including the numbers, disciplines, and grading of staff, the nature/duration of their involvement, and use of volunteers will be collected to comprehensively characterise different models of palliative care provision in the participating sites. With findings from this step, a new categorical variable will be created representing 'model of care' to be included in our analysis.

Sample size

In the development phase, based on standard recommendations for fitting multivariate models, a minimum of 50+8×m (where m is the number of predictors) is required to test the hypothesis that the population multiple correlation equals zero with a power of 80%, alpha = 5% and a medium effect size for the regression analysis (R2=0.13).[46] We estimated a sample size of 450 episodes per

For peer review only - http://bmjopen.bmj.com/site/about/guidelines.xhtml

setting (allowing for 25% incomplete episodes and up to 10% of complete episodes being cost outliers with unusually high or low costs). A total of 1,350 patient episodes across settings is needed.

In the validation phase, again a minimum of 50+8×m (where m is the number of predictors) is required to test the hypothesis that the population multiple correlation equals zero with a power of 80%, alpha = 5% and a medium effect size for the regression analysis (R2=0.13).[46] In Phase II, the number of predictors reflects the casemix variables, and needs to be clinically relevant[47] to ensure a meaningful case-mix classification. We expect the final casemix classification to contain 5-8 casemix variables, based on the Australian experience.[13] We have estimated that data from 114 (50 + (8x8)) episodes of care per setting (hospice, hospital, community) are needed. Assuming an average of three episodes per participant[15] in this longitudinal phase, this represents at least 38 participants per setting. Allowing for 50% attrition, we estimate needing a total of 228 participants, which will provide about 684 patient episodes. The sample size will be re-calculated and inflated using the design effect based on intraclass correlation which will be estimated through interim analyses. A total of 300 participants across settings is needed, including extra 25% participants allowing for intraclass correlation.

Missing data and data handling

Data will be collected prospectively and recorded on an electronic database. Any data transferred from the sites to the central study database will be carried out under the NHS Code of Practice on Confidentiality. Data will be cleaned and cross-checked using a number of internal checks (automated data validation on entry, independent cross-checking of a 5% sample of data from each site, and range checking across all data types) to track errors and inconsistencies and amend where possible. Checked data will be transferred to statistical software (Stata SE v12) for analysis. Missing Value Analysis will be used to quantify missing values and understand the reason for missing data: drop-out of participants, errors in data entry, and missing with no identifiable reasons. We will adopt multiple imputation technique based on Markov Chain Monte Carlo (MCMC) techniques if the percentage of observed missing values in independent variables exceeds 10% provided values are missing at random, and carry out sensitivity analysis for effects on casemix classification, predictive validity, and misclassification, where feasible.

Statistical analysis methods

We will use descriptive statistics including frequencies, mean and standard deviation; and median and interquartile ranges as appropriate to describe the sample characteristics, and number and

For peer review only - http://bmjopen.bmj.com/site/about/guidelines.xhtml

length of episodes of care. Trajectory of outcomes and costs will be described, comparing patterns in cancer and non-cancer patients, and contrasting those with no unmet needs and those with unmet needs. We will compare our data with existing phase and episode data from the Australian Palliative Care Outcome Collaboration.[48] In the Australian classification, five casemix criteria (phase of illness, problem severity, functional status and dependency, age, model of care) were found to be most strongly predictive of resource use. If our analyses demonstrate the same five criteria as most predictive of resource use, the Australian classification or a refinement of it will be adopted. If there is disparity, then the data on individual casemix criteria will be used to develop a new casemix classification, using a recursive partitioning approach – specifically classification and regression trees (CART)[49, 50] compared to multivariable regression (Figure 1).

Figure 1 Data analysis flowchart here

Qualitative analysis methods

Audio-recordings will be transcribed verbatim and checked for accuracy and entered into NVivo 10. Data will be independently coded and analysed by two members of the research team. We will adopt a similar approach to Pinnock,[51] undertaking a thematic and narrative analysis of interviews, exploring how perspectives evolve over time, with detailed attention to patient and family perspectives on experience of care in each setting and transitions, including potential interventions (and hence cost levers or other triggers) to influence changes in settings of care.

Model development

The distribution of casemix criteria (cost predictors) in the participant population will be described based on Phase of Illness, functional status and problem severity, using parametric or non-parametric statistics, as appropriate. In order to reduce the number of cost predictors, we will initially use univariate analysis to explore the association and strength of association between casemix criteria and cost of the episode of care (quantifying cost by per diem cost, per phase cost and total cost of episode). We will use generalised linear model (multivariate regression model) to select variables that will then be applied in a hierarchical manner to form a branching classification in which each cost driver is incorporated only once.

We will select variables according to how much of the predictive error (R-square) each variable predicts and the related p-value. After each bootstrap iteration we will rank the variables according

For peer review only - http://bmjopen.bmj.com/site/about/guidelines.xhtml

to these two criteria and remove the weakest (non-significant) predictor. Variables shown in Table 2 and 3 and the newly created categorical variable representing 'model of care' will be initially entered into the models and then non-significant predictors removed at each iteration using a Bootstrap sub-sampling strategy. We will create dummy variables to enable each level of Phase of Illness and Model of Care (categorical variables) to be assessed individually. Internal validation will be conducted by using such methods as bootstrapping or cross-validation to quantify any optimism in the predictive performance (calibration and discrimination) of the developed classification.

Model specification

The casemix classification for each outcome across three settings will be presented including all regression coefficients and model intercept (Figure 2). How to use the classification will be explained. Understanding how casemix variables predict cost of the episode of care enables a robust casemix classification to be developed. But in order for casemix adjustment to occur, there also needs to be a good understanding of how the casemix variables impact on clinical outcomes, as well as resource use. This casemix adjustment methodology is described in the recently published Department of Health document,[52] which recommends that significant casemix variables are identified, and that the size of their relationship with clinical outcomes is determined in advance.

Multi-level modelling will be undertaken to ensure both of these steps to be delivered in this study by determining (1) which casemix criteria are most strongly associated with clinical outcomes, allowing for clustering at the level of 'participant' (where episodes occur in the same individual), at the level of 'model of care' (where care is received according to similar models), and at the level of 'site' (where care is delivered in a similar way within site), and (2) which casemix criteria are the strongest cost predictor (including per diem cost, per phase cost, and total episode cost).

Figure 2 Casemix classification specification here

Model performance

The performance of the casemix classification will be assessed in both the development and validation datasets. Classification measures including sensitivity, specificity, predictive values, and net reclassification improvement will be reported and cut points selected a-priori. We will primarily focus on discrimination for the classification development phase, and assess both discrimination and calibration for the validation phase.[53] Discrimination of the classification will be measured using the

For peer review only - http://bmjopen.bmj.com/site/about/guidelines.xhtml

For peer review only

concordance statistic and confidence intervals (c-statistic).[54] Calibration of the classification will be measured using calibration plot and Hosmer-Lemeshow goodness-of-fit test.[55]

Development vs. validation

Data will be collected from patients, caregivers, and clinicians during the validation phase to compare patient-reported problem severity i.e. 'felt' need collected in validation phase only (as measured by the patient-reported IPOS) and professional-reported problem severity i.e. 'normative' need (as measured by the clinician-reported IPOS, Phase of Illness, AKPS), by reporting correlation across levels of complexity and across conditions. We will assess how professional-reported measures relate to patient-reported outcomes across different levels of complexity (i.e. in relation to casemix classes), in order to determine optimal outcome measures and quality indicators.

We will compare data on resource use from two sources: i) clinician-completed brief PRUS, and ii) patients and family-completed Client Service Receipt Inventory, to better understand how the PRUS maps to patients' receipt of resources, and evaluate which generic services are utilised across different providers, regions, and the context of different models of specialist palliative care.

Ethical considerations

The study protocol and documents (e.g., the participant information sheet, consent and declaration form) have been approved by the NHS Health Research Authority London – Camberwell St Giles Research Ethics committee (15/LO/0887) for the development phase, and NHS Health Research Authority London – Bromley Research Ethics committee (16/LO/1021) for the validation phase.

All eligible participants are fully informed before consent is sought by local research team or research nurses or project research team through the information sheets and verbal explanation on the aims and methods of the study and procedures that might be involved. Participants can withdraw at any time up to analysis of their data, without giving any reason. It is possible that participants may become distressed or raise issues during this study which raise concerns or warrant a change in their medical management, but we do not expect the questionnaires will themselves cause distress so much as uncover pre-existing distress which has not been acknowledged or recognised. Should this be the case, then our distress protocol will be followed. We anticipate distress will be infrequent, given the general nature of the questionnaires. It is likely that any distress will reflect advanced disease and experiences of care, and not the questionnaires themselves. All of

For peer review only

For peer review only - http://bmjopen.bmj.com/site/about/guidelines.xhtml

the study team members have completed Good Clinical Practice training, and specific training on addressing distress in palliative care.

Our existing Patient/Public Advisory Group and extended Consumer Panel have been and will continue to be consulted throughout the study to ensure that the study is carried out in an ethical and respectful way, and has the highest possible relevance and benefit to patients and families. A Project Steering Committee meets six monthly to monitor recruitment, review the detailed progress of the study, and make recommendations for overall direction and strategy.

Dissemination

This study will lead to patient benefit through improved matching of resources to needs at individual patient-level and will better enable the NHS to deliver high quality, patient-centred palliative care in last year of life. The results of the study will be published in peer-reviewed publications and will also be presented at national and international conferences.

For peer review only

For peer review only - http://bmjopen.bmj.com/site/about/guidelines.xhtml

Acknowledgements

This article presents independent research C-CHANGE funded by the National Institute for Health Research (NIHR) under the Programme Grants for Applied Research scheme (RP-PG-1210-12015) and the NIHR CLAHRC funding scheme, through the Collaboration for Leadership in Applied Health Research and Care (CLAHRC) in South London. CLAHRC South London is part of the NIHR and is a partnership between King's Health Partners, St George's, University of London, and St George's Healthcare NHS Trust. The C-CHANGE research team acknowledges the support of the National Institute of Health Research Clinical Research Network (NIHR CRN).

Contributors

PG drafted the study protocol with input from all authors. MD, JMD and AD provided statistical advice and support. AMF, SMO, CP, SP and PG contributed to the acquisition and interpretation of data for the work. IJH and FEM conceived the study and were in charge of overall direction and planning. KE provided experience from the Australian palliative care casemix development.[13] FEM is the Chief Investigator and designed the C-CHANGE research programme of which this is part. All authors provided critical feedback, helped revise the manuscript, and are accountable for the accuracy and integrity of all aspects of the work.

Funding

This work was supported by NIHR Programme Grants for Applied Research (Grant number RP-PG-1210-12015).

Disclaimer

The views and opinions expressed are those of the authors and do not necessarily reflect those of the NHS, the NIHR, MRC, CCF, NETSCC, the NIHR Programme Grants for Applied Research, or the Department of Health.

Competing interests

None declared.

Ethics approval

The NHS Health Research Authority (HRA) Approval.

For peer review only

For peer review only - http://bmjopen.bmj.com/site/about/guidelines.xhtml

References

1. Currow DC, Abernethy AP and Fazekas BS. Specialist palliative care needs of whole populations: a feasibility study using a novel approach. *Palliative Medicine*. 2004; 18: 239-47.
2. Fitch MI. Needs of patients living with advanced disease. *Canadian Oncology Nursing Journal* 2005; 15: 230-42.
3. Department of Health. Assessing health care needs. London: National Health Service Management Executive, 1991.
4. The Economist Intelligence Unit. The 2015 Quality of Death Index: Ranking palliaitve care across the world. 2015.
5. Sleeman KE, Davies JM, Verne J, Gao W and Higginson IJ. The changing demographics of inpatient hospice death: Population-based cross-sectional study in England, 1993–2012. *Palliative Medicine*. 2016; 30: 45-53.
6. Rosenwax L, Spilsbury K, McNamara BA and Semmens JB. A retrospective population based cohort study of access to specialist palliative care in the last year of life: who is still missing out a decade on? *BMC Palliative Care*. 2016; 15: 46.
7. Hughes-Hallett T, Craft A, Davies C, Mackay I and Nielsson T. Funding the right care and support for everyone: Creating a fair and transparent funding system; the final report of the palliative care funding review. *Palliative Care Funding Review*. 2011.
8. Lancaster H, Finlay I, Downman M and Dumas J. Commissioning of specialist palliative care services in England. *BMJ Supportive & Palliative Care*. 2017.
9. Etkind SN, Bone AE, Gomes B, et al. How many people will need palliative care in 2040? Past trends, future projections and implications for services. *BMC medicine*. 2017; 15: 102.
10. Lee LA, Eagar KM and Smith MC. Subacute and non-acute casemix in Australia. *The Medical Journal of Australia*. 1998; 169: 22-5.
11. Eagar K, Cromwell D, Kennedy C and Lee L. Classifying sub-acute and non-acute patients: Results of the New South Wales Casemix Area Network study. *Australian Health Review*. 1997; 20: 26-42.
12. Turner-Stokes L, Sutch S, Dredge R and Eagar K. International casemix and funding models: lessons for rehabilitation. *Clinical rehabilitation*. 2011; 26: 195-208.
13. Eagar K, Gordon R, Hodkinson A, et al. The Australian National Sub-Acute and Non-Acute Patient Classification (AN-SNAP): report of the National Sub-Acute and Non-Acute Casemix Classification Study. Centre for Health Service Development, University of Wollongong1997.
14. Eagar K, Green J and Gordon R. An Australian casemix classification for palliative care: technical development and results. *Palliative Medicine*. 2004; 18: 217-26.
15. Eagar K, Watters P, Currow DC, Aoun SM and Yates P. The Australian Palliative Care Outcomes Collaboration (PCOC)--measuring the quality and outcomes of palliative care on a routine basis. *Australian Health Review*. 2010; 34: 186-92.
16. Eagar K, Gordon R, Green J and Smith M. An Australian casemix classification for palliative care: lessons and policy implications of a national study. *Palliative Medicine*. 2004; 18: 227-33.
17. Collins GS, Reitsma JB, Altman DG and Moons KM. Transparent reporting of a multivariable prediction model for individual prognosis or diagnosis (tripod): The tripod statement. *Annals of Internal Medicine*. 2015; 162: 55-63.
18. Hinds J. *National Survey of Patient Activity Data for Specialist Palliative Care Services: Minimum Data Set (MDS) Full Report for the Year 2014-2015*. London: The National Council for Palliative Care, 2016.
19. Castro FG, Kellison JG, Boyd SJ and Kopak A. A methodology for conducting integrative mixed methods research and data analyses. *Journal of Mixed Methods Research*. 2010; 4: 342-60.
20. Hjermstad M, Loge JH and Kaasa S. Methods for assessment of cognitive failure and delirium in palliative care patients: implications for practice and research. *Palliative Medicine*. 2004; 18: 494-506.

For peer review only - http://bmjopen.bmj.com/site/about/guidelines.xhtml

21. Radbruch L, Sabatowski R, Loick G, et al. Cognitive impairment and its influence on pain and symptom assessment in a palliative care unit: development of a Minimal Documentation System. *Palliative Medicine*. 2000; 14: 266-76.
22. Higginson IJ, McCrone P, Hart SR, Burman R, Silber E and Edmonds PM. Is Short-Term Palliative Care Cost-Effective in Multiple Sclerosis? A Randomized Phase II Trial. *Journal of Pain and Symptom Management*. 2009; 38: 816-26.
23. Bausewein C, Booth S, Gysels M, Kühnbach R, Haberland B and Higginson IJ. Individual breathlessness trajectories do not match summary trajectories in advanced cancer and chronic obstructive pulmonary disease: results from a longitudinal study. *Palliative Medicine*. 2010; 24: 777-86.
24. Buller T. Advance consent, critical interests and dementia research. *Journal of Medical Ethics*. 2015; 41: 701-7.
25. Mather H, Guo P, Firth A, et al. Phase of Illness in palliative care: Cross-sectional analysis of clinical data from community, hospital and hospice patients. *Palliative Medicine*. DOI: 10.1177/0269216317727157.
26. Masso M, Allingham SF, Banfield M, et al. Palliative Care Phase: Inter-rater reliability and acceptability in a national study. *Palliative Medicine*. 2015; 29: 22-30.
27. Coventry PA, Grande GE, Richards DA and Todd CJ. Prediction of appropriate timing of palliative care for older adults with non-malignant life-threatening disease: a systematic review. *Age and Ageing*. 2005; 34: 218-27.
28. Franks PJ, Salisbury C, Bosanquet N, et al. The level of need for palliative care: a systematic review of the literature. *Palliative Medicine*. 2000; 14: 93-104.
29. Luddington L, Cox S, Higginson I and Livesley B. The need for palliative care for patients with non-cancer diseases: a review of the evidence. *International Journal of Palliative Nursing*. 2001; 7: 221-6.
30. Tebbit P. *Population-based Needs Assessment for Palliaitve and End of Life Care*. London: National Council for Palliative Care, 2008.
31. Drummond M, Weatherly H and Ferguson B. *Economic evaluation of health interventions*. 2008.
32. Kirch W. *Encyclopedia of Public Health*. Dordrecht: Springer Netherlands, 2008, p.267.
33. Curtis L. *Unit costs of health and social care 2014*. Canterbury: Personal Social Services Research Unit, University of Kent, 2014.
34. *Joint Formulary Committe. British National Formulary (BNF) 68*. London: BMJ Publishing Group Ltd and Royal Pharmaceutical Society, 2014.
35. Department of Health. NHS reference costs 2013 to 2014. 2014.
36. Wolff J, McCrone P, Patel A, Auber G and Reinhard T. A time study of physicians' work in a German University Eye Hospital to estimate unit costs. *PLOS ONE*. 2015; 10: e0121910.
37. Sutherland JM. Pricing hospital care: Global budgets and marginal pricing strategies. *Health Policy*. 2015; (in press).
38. Tan SS, Bakker J, Hoogendoorn ME, et al. Direct cost analysis of Intensive Care Unit stay in four European countries: Applying a standardized costing methodology. *Value in Health*. 2012; 15: 81-6.
39. Department of Health. NHS costing manual. 2012.
40. Abernethy AP, Shelby-James T, Fazekas BS, Woods D and Currow DC. The Australia-modified Karnofsky Performance Status (AKPS) scale: a revised scale for contemporary palliative care clinical practice [ISRCTN81117481]. *BMC Palliative Care*. 2005; 4: 7.
41. Collin C, Wade DT, Davies S and Horne V. The Barthel ADL Index: a reliability study. *International disability studies*. 1988; 10: 61-3.
42. King J, Gibbons E, Graham C and Walsh J. Developing measures of people's self-reported experiences of integrated care. *Picker Institute Europe and University of Oxford*. 2013.

For peer review only

For peer review only - http://bmjopen.bmj.com/site/about/guidelines.xhtml

43. Koffman J, Higginson IJ, Hall S, Riley J, McCrone P and Gomes B. Bereaved relatives' views about participating in cancer research. *Palliative Medicine*. 2012; 26: 379-83.
44. Addington-Hall J and McPherson C. After-death interviews with surrogates/bereaved family members: Some issues of validity. *Journal of Pain and Symptom Management*. 2001; 22: 784-90.
45. Costantini M, Beccaro M and Merlo F. The last three months of life of Italian cancer patients. Methods, sample characteristics and response rate of the Italian Survey of the Dying of Cancer (ISDOC). *Palliative Medicine*. 2005; 19: 628-38.
46. VanVoorhis CW and Morgan BL. Understanding power and rules of thumb for determining sample sizes. *Tutorials in Quantitative Methods for Psychology*. 2007; 3: 43-50.
47. Ogundimu EO, Altman DG and Collins GS. Adequate sample size for developing prediction models is not simply related to events per variable. *Journal of clinical epidemiology*. 2016; 76: 175-82.
48. Currow DC, Allingham S, Yates P, Johnson C, Clark K and Eagar K. Improving national hospice/palliative care service symptom outcomes systematically through point-of-care data collection, structured feedback and benchmarking. *Supportive care in cancer : official journal of the Multinational Association of Supportive Care in Cancer*. 2015; 23: 307-15.
49. Gao J, Johnston GM, Lavergne MR and McIntyre P. Identifying population groups with low palliative care program enrolment using classification and regression tree analysis. *Journal of palliative care*. 2011; 27: 98-106.
50. Lemon SC, Roy J, Clark MA, Friedmann PD and Rakowski W. Classification and regression tree analysis in public health: Methodological review and comparison with logistic regression. *ann behav med*. 2003; 26: 172-81.
51. Pinnock H, Kendall M, Murray SA, et al. Living and dying with severe chronic obstructive pulmonary disease: multi-perspective longitudinal qualitative study. *BMJ*. 2011; 342.
52. Department of Health. Patient reported outcome measures (PROMs) in England: the case-mix adjustment methodology. 2012.
53. Steyerberg EW, Vickers AJ, Cook NR, et al. Assessing the performance of prediction models: a framework for some traditional and novel measures. *Epidemiology (Cambridge, Mass)*. 2010; 21: 128-38.
54. Cook NR. Statistical Evaluation of Prognostic versus Diagnostic Models: Beyond the ROC Curve. *Clinical Chemistry*. 2007; 54: 17.
55. Austin PC and Steyerberg EW. Graphical assessment of internal and external calibration of logistic regression models by using loess smoothers. *Statistics in Medicine*. 2014; 33: 517-35.

For peer review only

For peer review only - http://bmjopen.bmj.com/site/about/guidelines.xhtml

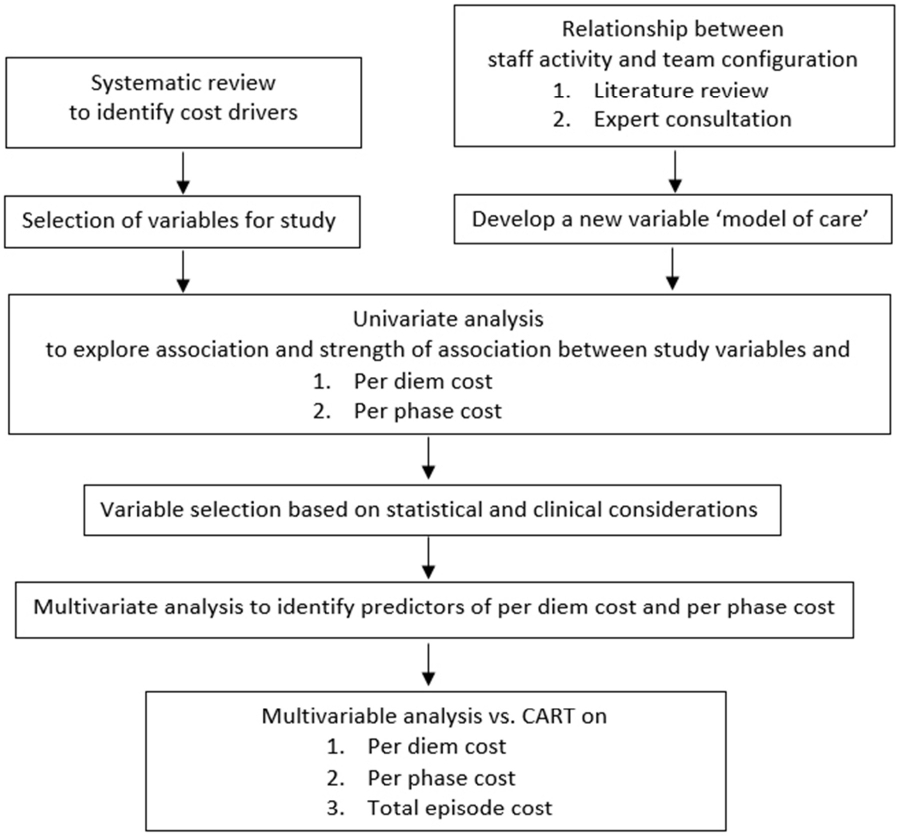

Figure 1 Data analysis flowchart

Figure 1 Data analysis flowchart

61x56mm (300 x 300 DPI)

nly

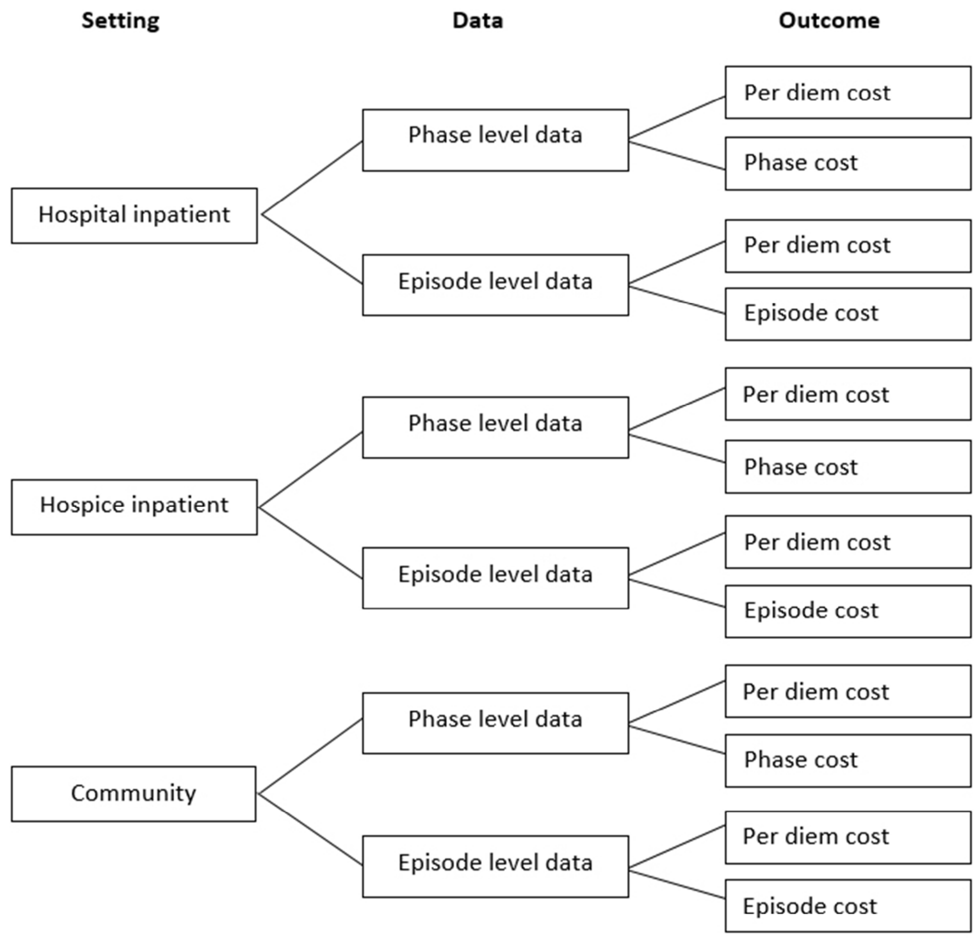

Figure 2 Casemix classification specification

Figure 2 Casemix classification specification

58x59mm (300 x 300 DPI)

TRIPOD Checklist: Prediction Model Development and Validation

Section/Topic	Item		Checklist Item	Page
**Title and abstract**				
Title	1	D;V	Identify the study as developing and/or validating a multivariable prediction model, the target population, and the outcome to be predicted.	1
Abstract	2	D;V	Provide a summary of objectives, study design, setting, participants, sample size, predictors, outcome, statistical analysis, results, and conclusions.	2
**Introduction**				
Background and objectives	3a	D;V	Explain the medical context (including whether diagnostic or prognostic) and rationale for developing or validating the multivariable prediction model, including references to existing models.	4-5
	3b	D;V	Specify the objectives, including whether the study describes the development or validation of the model or both.	4-5
**Methods**				
Source of data	4a	D;V	Describe the study design or source of data (e.g., randomized trial, cohort, or registry data), separately for the development and validation data sets, if applicable.	5-6
	4b	D;V	Specify the key study dates, including start of accrual; end of accrual; and, if applicable, end of follow-up.	5-6
Participants	5a	D;V	Specify key elements of the study setting (e.g., primary care, secondary care, general population) including number and location of centres.	6-7
	5b	D;V	Describe eligibility criteria for participants.	6-7
	5c	D;V	Give details of treatments received, if relevant.	6-7
Outcome	6a	D;V	Clearly define the outcome that is predicted by the prediction model, including how and when assessed.	7-9
	6b	D;V	Report any actions to blind assessment of the outcome to be predicted.	N/A
Predictors	7a	D;V	Clearly define all predictors used in developing the multivariable prediction model, including how and when they were measured.	9-11
	7b	D;V	Report any actions to blind assessment of predictors for the outcome and other predictors.	N/A
Sample size	8	D;V	Explain how the study size was arrived at.	11-12
Missing data	9	D;V	Describe how missing data were handled (e.g., complete-case analysis, single imputation, multiple imputation) with details of any imputation method.	12
Statistical analysis methods	10a	D	Describe how predictors were handled in the analyses.	12-14
	10b	D	Specify type of model, all model-building procedures (including any predictor selection), and method for internal validation.	12-16
	10c	V	For validation, describe how the predictions were calculated.	12-16
	10d	D;V	Specify all measures used to assess model performance and, if relevant, to compare multiple models.	12-16
	10e	V	Describe any model updating (e.g., recalibration) arising from the validation, if done.	12-16
Risk groups	11	D;V	Provide details on how risk groups were created, if done.	12-16
Development vs. validation	12	V	For validation, identify any differences from the development data in setting, eligibility criteria, outcome, and predictors.	16
**Results**				
Participants	13a	D;V	Describe the flow of participants through the study, including the number of participants with and without the outcome and, if applicable, a summary of the follow-up time. A diagram may be helpful.	N/A
	13b	D;V	Describe the characteristics of the participants (basic demographics, clinical features, available predictors), including the number of participants with missing data for predictors and outcome.	N/A
	13c	V	For validation, show a comparison with the development data of the distribution of important variables (demographics, predictors and outcome).	N/A
Model development	14a	D	Specify the number of participants and outcome events in each analysis.	N/A
	14b	D	If done, report the unadjusted association between each candidate predictor and outcome.	N/A
Model specification	15a	D	Present the full prediction model to allow predictions for individuals (i.e., all regression coefficients, and model intercept or baseline survival at a given time point).	N/A
	15b	D	Explain how to use the prediction model.	N/A
Model performance	16	D;V	Report performance measures (with CIs) for the prediction model.	N/A
Model-updating	17	V	If done, report the results from any model updating (i.e., model specification, model performance).	N/A
**Discussion**				
Limitations	18	D;V	Discuss any limitations of the study (such as nonrepresentative sample, few events per predictor, missing data).	12
Interpretation	19a	V	For validation, discuss the results with reference to performance in the development data, and any other validation data.	N/A
	19b	D;V	Give an overall interpretation of the results, considering objectives, limitations, results from similar studies, and other relevant evidence.	N/A
Implications	20	D;V	Discuss the potential clinical use of the model and implications for future research.	15-16
**Other information**				
Supplementary information	21	D;V	Provide information about the availability of supplementary resources, such as study protocol, Web calculator, and data sets.	N/A
Funding	22	D;V	Give the source of funding and the role of the funders for the present study.	18

*Items relevant only to the development of a prediction model are denoted by D, items relating solely to a validation of a prediction model are denoted by V, and items relating to both are denoted D;V. We recommend using the TRIPOD Checklist in conjunction with the TRIPOD Explanation and Elaboration document.

For peer review only - http://bmjopen.bmj.com/site/about/guidelines.xhtml

BMJ Open

Development and validation of a casemix classification to predict costs of specialist palliative care provision across inpatient hospice, hospital, and community settings in the UK: A study protocol

Journal:	*BMJ Open*
Manuscript ID	bmjopen-2017-020071.R2
Article Type:	Protocol
Date Submitted by the Author:	06-Feb-2018

Complete List of Authors:	Guo, Ping; King's College London, Cicely Saunders Institute, Department of Palliative Care, Policy and Rehabilitation<br>Dzingina, Mendwas; King's College London, Cicely Saunders Institute, Department of Palliative Care, Policy and Rehabilitation<br>Firth, Alice; King's College London, Cicely Saunders Institute, Department of Palliative Care, Policy and Rehabilitation<br>Davies, Joanna; King's College London, Cicely Saunders Institute, Department of Palliative Care, Policy and Rehabilitation<br>Douiri, Abdel; King's College London, Primary Care And Public Health Sciences<br>O'Brien, Suzanne; King's College London, Cicely Saunders Institute, Department of Palliative Care, Policy and Rehabilitation<br>Pinto, Cathryn; King's College London, Cicely Saunders Institute, Department of Palliative Care, Policy and Rehabilitation<br>Pask, Sophie; King's College London, Cicely Saunders Institute, Department of Palliative Care, Policy and Rehabilitation<br>Higginson, Irene; Kings College London, Cicely Saunders Institute, Department of Palliative Care, Policy and Rehabilitation<br>Eagar, Kathy; University of Wollongong, Australian Health Services Research Institute, Centre for Health Service Development<br>Murtagh, Fliss; University of Hull, Wolfson Palliative Care Research Centre, Hull York Medical School

**Primary Subject Heading**:	Palliative care
Secondary Subject Heading:	Health services research
Keywords:	Adult palliative care < PALLIATIVE CARE, Casemix classification, Validation, Cost predictors

SCHOLARONE™ Manuscripts

TITLE PAGE

Full title:

Development and validation of a casemix classification to predict costs of specialist palliative care provision across inpatient hospice, hospital, and community settings in the UK: A study protocol

Author details:

Dr Ping Guo,
Project Manager and Research Associate
Cicely Saunders Institute, Department of Palliative Care, Policy & Rehabilitation, King's College London, Bessemer Road, SE5 9PJ, UK
Correspondence to P. Guo: Email: ping.guo@kcl.ac.uk Telephone: 020 7848 5523

Dr Mendwas Dzingina, Cicely Saunders Institute, Department of Palliative Care, Policy & Rehabilitation, King's College London, Bessemer Road, SE5 9PJ, UK

Alice M Firth, Cicely Saunders Institute, Department of Palliative Care, Policy & Rehabilitation, King's College London, Bessemer Road, SE5 9PJ, UK

Joanna M Davies, Cicely Saunders Institute, Department of Palliative Care, Policy & Rehabilitation, King's College London, Bessemer Road, SE5 9PJ, UK

Dr Abdel Douiri, Department of Primary Care and Public Health Sciences, Addison House, Guy's Campus, King's College London, UK

Suzanne M O'Brien, Cicely Saunders Institute, Department of Palliative Care, Policy & Rehabilitation, King's College London, Bessemer Road, SE5 9PJ, UK

Cathryn Pinto, Cicely Saunders Institute, Department of Palliative Care, Policy & Rehabilitation, King's College London, Bessemer Road, SE5 9PJ, UK

Sophie Pask, Cicely Saunders Institute, Department of Palliative Care, Policy & Rehabilitation, King's College London, Bessemer Road, SE5 9PJ, UK

Professor Irene J Higginson, Cicely Saunders Institute, Department of Palliative Care, Policy & Rehabilitation, King's College London, Bessemer Road, SE5 9PJ, UK

Professor Kathy Eagar, University of Wollongong, Australian health Services Research Institute, Centre for Health Service Development, Wollongong, Australia

Professor Fliss EM Murtagh, Wolfson Palliative Care Research Centre, Hull York Medical School, University of Hull, UK

For peer review only - http://bmjopen.bmj.com/site/about/guidelines.xhtml

Development and validation of a casemix classification to predict costs of specialist palliative care provision across inpatient hospice, hospital, and community settings in the UK: A study protocol

ABSTRACT

Introduction: Provision of palliative care is inequitable with wide variations across conditions and settings in the UK. Lack of a standard way to classify by case complexity is one of the principle obstacles to addressing this. We aim to develop and validate a casemix classification to support the prediction of costs of specialist palliative care provision.

Methods and analysis:

Phase I - A cohort study to determine the variables and potential classes to be included in a casemix classification. Data are collected from clinicians in palliative care services across inpatient hospice, hospital, and community settings on: patient demographics, potential complexity/casemix criteria, and patient-level resource use. Cost predictors are derived using multivariate regression and then incorporated into a classification using Classification and Regression Trees (CART). Internal validation will be conducted by bootstrapping to quantify any optimism in the predictive performance (calibration and discrimination) of the developed classification.

Phase II - A mixed methods cohort study across settings for external validation of the classification developed in Phase I. Patient and family caregiver data will be collected longitudinally on demographics, potential complexity/casemix criteria, and patient-level resource use. This will be triangulated with data collected from clinicians on potential complexity/casemix criteria and patient-level resource use, and with qualitative interviews with patients and caregivers about care provision across difference settings. The classification will be refined on the basis of its performance in the validation data set.

Ethics and dissemination: The study has been approved by the National Health Service (NHS) Health Research Authority Research Ethics Committee. The results are expected to be disseminated in 2018 through papers for publication in major palliative care journals; policy briefs for clinicians, commissioning leads, and policy makers; and lay summaries for patients and public.

Study registration number: ISRCTN90752212

For peer review only - http://bmjopen.bmj.com/site/about/guidelines.xhtml

Strengths and limitations of this study

- This is the first study in the UK to determine the variables and potential classes to be included in a casemix classification, which predicts costs of specialist palliative care provision across inpatient hospice, hospital, and community settings.
- Transparent reporting of a multivariable prediction model for individual prognosis or diagnosis (TRIPOD) is used to guide the reporting of this study.
- The newly developed casemix classification in Phase I will be externally validated by using a different dataset in Phase II.
- This study also promotes the implementation of outcome measures in palliative care into routine clinical practice across different settings of care.
- This is a UK focused study so the casemix classification may not be directly applied to palliative care in other countries without further investigation and refinement.

For peer review only

INTRODUCTION

People with advanced and incurable illness often suffer complex and multiple symptoms and psychosocial concerns because of their illness or impending death.[1, 2] Their families may provide day-to-day care, as well as being affected by their own anxieties, concerns, and potential losses. These bring increased need for health and social care, with need being defined as 'the ability to benefit from health or social care interventions'.[3] Palliative care has developed to meet the needs of these patients and families, which addresses physical/psychological symptoms and provides social, practical and spiritual support. The UK ranks first in the 2015 Quality of Death Index - a measure of the quality of dying in 80 countries,[4] and the hospice movement in the UK has provided a model of good palliative care for those in need.

However, marked inequities exist in provision of palliative care across England. Older people and those with non-cancer diagnoses are less likely to access specialist palliative care.[5, 6] There are also major geographical variations, ranging from £186 to £6,213 per person across different Primary Care Trusts in 2010,[7, 8] often resulting in a poor match between individual needs, resources provided to meet those needs, and health outcomes achieved. With an ageing population and increasing rates of chronic diseases, the growing healthcare burden is overwhelmingly challenging in terms of health resource allocation around the UK.[9] With recognition of the constraints on resources, there is support for the systematic approach to mapping individual needs accurately and improving the quality and efficiency of palliative care. This has been endorsed as a high priority nationally.[7]

The Diagnosis Related Group is a useful classification of healthcare needs driven by the diagnosis but is inappropriate for palliative care, because palliative care needs are not driven by the diagnosis but by factors such as functional status and symptoms. Palliative care needs a consistent method of classifying types of patients with complexity of needs, treatment, and costs, using casemix criteria.[10-12] It is necessary to identify those with more complex palliative needs, requiring more resources. An Australian casemix classification for palliative care was developed in 1997, empirically tested and progressively refined over time.[13-15] The Australian casemix classification consists of classes defined by five criteria including Phase of Illness, problem severity, functional status and dependency, age, model of care, as most strongly predictive of resource use.[16] Full class definition and categorisation is available at http://www.pcoc.org.au/. Its implementation proved the possibility of consistently and routinely collecting data in practice in Australia.[16] However, due to variations in outcome measures collected and palliative care provided between countries, it is unclear whether any existing palliative care classification can be easily applied to the UK to address unmet needs and resolve the inequity.

For peer review only - http://bmjopen.bmj.com/site/about/guidelines.xhtml

This study is part of a five year NIHR funded C-CHANGE programme (RP-PG-1210-12015). It aims to develop and validate a casemix classification for palliative care in the UK. Specific objectives are:

- To determine the cost predictors of specialist palliative care, adjusting for important confounding factors including unmet needs.
- To develop and validate a casemix classification for specialist palliative care.

METHODS AND ANALYSIS

The Transparent Reporting of a multivariable prediction model for Individual Prognosis Or Diagnosis (TRIPOD) Statement [17] is used to guide the reporting of this study protocol.

Study design and source of data

This study consists of two phases: Development phase (Phase I) and validation phase (Phase II). In the analysis part of Phase I, we will identify variables that predict costs to be included in casemix classification through a cohort study with only clinician data, and develop potential casemix classes for palliative care which will be tested in individual episodes of care (internal validation). In Phase II, we will prospectively validate these potential classes in a new cohort study with patient, caregiver, and clinician data, and include qualitative interviews to ensure it works during care transitions and longitudinally over the course of illness (external validation).

Phase I – Development phase

This is a cohort study to determine the variables and potential classes to be included in a casemix classification, based on individual episodes of care across inpatient (hospice and hospital) and community settings. An 'episode of care' starts when a patient is admitted to inpatient services or begins to receive specialist palliative care from a community-based or outpatient service, and ends when a patient is discharged from that service or dies. The median duration of an episode of care is expected to be under 14 days in inpatient and at a median of 72 days in community settings.[18]

Phase I was conducted between 31st July 2015 and 30th September 2016 and follow-up ended 30th November 2016. Data were collected from clinicians through surveys, including demographic/clinical data, episode start/end data, patient attributes that predict palliative care resource consumption (e.g., Phase of Illness, functional status, and problem severity as identified in the Australian casemix classification study [13, 14, 16]), plus information on patient-level resource use (staff activity and clinical services) in specialist palliative care settings.

For peer review only - http://bmjopen.bmj.com/site/about/guidelines.xhtml

Phase II – Validation phase

This phase is a mixed methods cohort study with a concurrent nested design:[19]

- Quantitative main component - Prospectively collect data from patients, caregivers and clinicians on palliative care needs, concerns, outcomes, and resources use.
- Qualitative nested component - Longitudinal interviews with a subsample of participants to understand care provision in each setting and transitions between settings.

Phase II will be conducted between 1st November 2016 and 31st December 2017, with follow-up ending on 28th February 2018. The same variables and measurements as Phase I will be collected from clinicians. Additionally, patient/caregiver participants will provide data on symptoms/concerns, experience of care, and their use of services, and be followed through all episodes of care from recruitment to death or the end of this study. A post bereavement survey will be conducted with caregivers where appropriate to identify symptoms/concerns immediately prior to death and support needs after death.

Participants

Phase I – Development phase

All adults (≥18 years) receiving specialist palliative care newly admitted in 10 participating sites during the study period (2 sites providing hospital advisory services, 1 providing community-based service only, 6 providing hospice inpatient and community-based services, 1 providing hospital advisory and community-based services) were included, regardless of primary diagnosis. We selected these sites with the aim of ensuring a representative sample in terms of population demographics (age distribution, ethnicity, socio-economic status, and rural/urban composition). Written informed consent was taken by a qualified member of the research or clinical team. This phase included individuals with limited, fluctuating, diminishing or lack of capacity, vital to ensure that any casemix classification is applicable to all palliative patients, not just those able to consent. It is recognised that a high proportion of those have impaired capacity, and these patients may need palliative care and resources most.[20-23] Therefore, if the clinician assessed that the patient did not currently have the capacity to give consent, assent was sought from an accompanying family member, or failing that, a staff member to whom the patient was known. Where a formally appointed Power of Attorney existed, this took precedence.

Phase II – Validation phase

For peer review only - http://bmjopen.bmj.com/site/about/guidelines.xhtml

All adults (≥18 years) receiving specialist palliative care newly admitted from 14 sites during the study period (participating sites in Phase I, but extending to four additional sites to increase recruitment) will be eligible, regardless of primary diagnosis. Additional sites were accepted if they could contribute to maintaining or extending representativeness in terms of population demographics (age distribution, ethnicity, socio-economic status, and rural/urban composition). Written informed consent will be sought from eligible patients (and their family caregivers) at the outset, including advance consent for study follow-up if capacity is lost. This study is of minimal risk with participants not exposed to undue harm.[24]

For both Phase I and II, a list of study sites can be obtained via: https://www.kcl.ac.uk/nursing/departments/cicelysaunders/research/studies/c-change/c-change.aspx.

Since transition of care is important for casemix, patient participants who transfer between settings (and caregiver participants) will be followed up. We will verbally confirm continuing consent with each participant when making such contact. If capacity of patient participants is lost, we will seek advice from a personal consultee on whether the patient should remain in the study to verify no change of decisions has been expressed by the patient prior to loss of capacity. On recruitment, participants will be asked if willing to be interviewed at a later stage. To capture variation in age, gender, diagnosis, and geographical location, we will purposively select 20-25 patients and family caregivers with at least two transitions of care for face-to-face interviews. Each semi-structured interview will last 40 minutes, but will be guided by each participant. In order to provide information on how care transitions might be better negotiated to improve outcomes and experiences, the interviews will cover communication, coordination of care, information/support needs, discharge planning, and experience of transitions.

Outcome

The outcome is cost of specialist palliative care (including per diem cost, per phase cost, and total episode cost) captured by: (1) staff activity matrix in both the development and validation phase, (2) the Palliative care Resource Use Score (PRUS) in both phases, and (3) Palliative Client Services Receipt Inventory (Pall-CSRI) in validation phase only.

Staff activity

For peer review only - http://bmjopen.bmj.com/site/about/guidelines.xhtml

For peer review only

At every contact, nurses, doctors, and allied health professionals will record the time spent on face-to-face and phone contacts, and patient-level administrative time per shift using the staff activity matrix paper version (Table 1). Staff training and site feedback of activity data were conducted regularly to improve and optimise data quality.

Table 1 Staff activity matrix (Each box is completed in units of 5 minutes, from 0 up to 120 minutes)

Staff time (mins)	Patient	Family/carer	Professional (internal)	Professional (external)
Face-to-face/phone time				
Administrative time				

Palliative care Resource Use Score (PRUS)

PRUS is a questionnaire specifically designed to capture palliative care resource use in a standardised way. It will be collected by staff at change of 'Phase of Illness'[25, 26] and at the end of each episode by recording the following information for the phase which has ended:

- Level of professional input (e.g., registered nurses, specialist palliative nursing staff, palliative doctors, and social workers) and whether this met patient/family needs.
- Level of 'out of hours' services by professional designation.
- Equipment, high cost drugs, diagnostic tests, and medical imaging. Within PRUS, the member of staff is asked to indicate whether or not equipment, high cost oral/transdermal medications, injectable medications/interventions, and medical imaging or tests were received by the participant, from a list derived from prior work to identify these options.
- Any unmet needs which provide insight into any gaps between needs and provision, as identified by a member of staff.[27-29] When completing PRUS, the member of staff is asked to estimate retrospectively if there were any unmet needs (Yes/No) in the care provided by health care assistants, registered nurses, palliative medicine doctors, and allied health care professionals. This member of staff is also asked whether there were any unmet needs in out of hours care, and whether the equipment provided met the patient's needs. If there were unmet needs identified in any of these areas above, a free text field is provided to specify these unmet needs.

Palliative Client Services Receipt Inventory (Pall-CSRI)

The Pall-CSRI is a patient/caregiver completed inventory of palliative care services received, and adapted from those used with palliative care populations.[30] It takes approximately 20 minutes to complete and collects retrospective information about the use of health/social care services, medication, living situation, income, employment and benefits, plus informal care. The Pall-CSRI will

For peer review only - http://bmjopen.bmj.com/site/about/guidelines.xhtml

be collected three monthly or at the end of each episode of care whichever is earlier in the validation phase.

In the development phase, only direct care costs will be calculated but not productivity losses.[31] Direct care cost refers to all costs due to resource use that are completely attributable to the use of a health care intervention or illness, which can be split into direct medical costs (the cost of a defined intervention and all follow-up costs for other medication and health care interventions in ambulatory, inpatient, nursing care, home, or other relevant settings) and direct non-medical costs (e.g., transportation costs and additional paid caregiver time).[32] In the validation phase, we will use the same health services costing perspective as in the development phase, but informal care costs are also considered to be in-scope and will be analysed separately, hypothesising that informal care costs are (1) greater in those with unmet needs, and (2) greater in those with non-cancer conditions. The costs of each resource item will be calculated by combining the resource use data with appropriate unit cost obtained from recognised sources including the annual compendium produced by the Personal Social Services Research Unit (PSSRU),[33] prices on the NHS supply chain website http://www.supplychain.nhs.uk, British national formulary,[34] and NHS reference costs.[35] The focus will be on staff time - the main resource in palliative care documented in a variety of settings and countries.[36-38] We will attribute costs according to a standard costing methodology adopted from the current NHS costing principles.[39]

Predictors (casemix criteria)

Proposed cost predictors can be categorised into three groups: (1) predictors collected from clinicians, (2) predictors collected from patients/family caregivers, and (3) model of care.

Predictors collected from clinicians

In both the development and validation phases, clinicians will record demographic, clinical and episode start administrative data. Episode end data will be collected at the end of each episode of care. Casemix data include Phase of Illness (stable, unstable, deteriorating, dying), functional status (measured by Australia-modified Karnofsky Performance Status - AKPS), dependency (Modified Barthel Index), problem severity (Integrated Palliative care Outcome Scale - IPOS staff version), and caregiver needs (Table 2). The measures are available direct from: Phase of Illness (www.pcoc.org.au), AKPS,[40] Modified Barthel Index,[41] and IPOS (www.pos-pal.org) respectively. Phase of Illness will be assessed daily for people receiving inpatient care and at each face-to-face contact basis for those receiving community-based care. Other variables including AKPS,

For peer review only - http://bmjopen.bmj.com/site/about/guidelines.xhtml

Modified Barthel Index, IPOS-staff (and caregiver needs when feasible) will be collected at the start of episode, end of episode, and at change of Phase of Illness.

Table 2 Data from clinicians in both development phase and validation phase

**Type of data**	**Proposed cost predictors**
Demographic data	Age
	Gender
	Postcode
	Ethnicity
	Marital status
	Living circumstances
	Need for interpreter
	Setting of care
Clinical data	Primary diagnosis
	Secondary diagnoses
	Co-morbidities
Episode start and end data	Episode start date
	Episode end date
	Endpoint of episode (discharged or died).
	Discharge destination, if discharged
Key casemix	Phase of Illness at start of episode
	Functional status (AKPS)
	Dependency (Modified Barthel Index)
	Problem severity (IPOS – staff version)
	Family/caregiver needs

For peer review only

Predictors collected from patients and family caregivers

Variables (Table 3) will be collected in the validation phase from patients/caregivers using face-to-face/telephone contacts and postal questionnaires according to their preferences. IPOS - patient version and Distress Thermometer will be collected from patients at the start of episode, at change of Phase of Illness, and at the end of episode. Views on Care (VoC) and SF-12v2 will be collected at the start and end of each episode of care. Selected questions capturing experiences of integrated care[42] will be collected at the end of episode only.

For peer review only - http://bmjopen.bmj.com/site/about/guidelines.xhtml

All caregiver reported measures will be collected at the start of episode, at change of Phase of Illness, and at the end of episode. Where phase length exceeds 4 weeks, data collected from patients (and caregivers) will be captured as a change of phase. If the patient is transferred to an out of scope inpatient setting, we will follow up with the patient when he or she gets home and retrospectively collect minimum information about this episode of care.

Table 3 Predictors collected from patients and family caregivers in validation phase only

**Data collection from patients**	**Data collection from family caregivers**
IPOS – patient version	Basic demographic information
Distress Thermometer	Distress Thermometer
Views on Care (VoC)	2 caregiver questions
SF-12v2*	Zarit 6 items
Patient experiences of integrated care	

* The measure could be completed by a family caregiver if the patient is too unwell to complete.

If the patient is deceased, data such as SF-12v2 and Pall-CSRI will be collected from the participating caregiver three months after death by postal questionnaire, along with the bereavement support information and contacts. The time between death and the postal questionnaire may influence caregivers' willingness to share their views; shorter periods possibly being too upsetting to contemplate involvement.[43] There is mixed evidence of the best time to make contact with potential participants for follow-back surveys[44, 45] and 3-4 months post bereavement is considered acceptable by bereaved families.[30]

Model of care

Models of care may be a stronger cost driver than patient-level variables.[13, 14, 16] Specialist palliative care services have various configurations of staff, interventions, and other characteristics. Currently, there is no consistent way to define models of palliative care. A separate, parallel study will be conducted in which service-level data including the numbers, disciplines, and grading of staff, the nature/duration of their involvement, and use of volunteers will be collected to comprehensively characterise different models of palliative care provision in the participating sites. With findings from this step, a new categorical variable will be created representing 'model of care' to be included in our analysis.

For peer review only - http://bmjopen.bmj.com/site/about/guidelines.xhtml

Sample size

In the development phase, based on standard recommendations for fitting multivariate models, a minimum of 50+8×m (where m is the number of predictors) is required to test the hypothesis that the population multiple correlation equals zero with a power of 80%, alpha = 5% and a medium effect size for the regression analysis (R2=0.13).[46] We estimated a sample size of 450 episodes per setting (allowing for 25% incomplete episodes and up to 10% of complete episodes being cost outliers with unusually high or low costs). A total of 1,350 patient episodes across settings is needed.

In the validation phase, again a minimum of 50+8×m (where m is the number of predictors) is required to test the hypothesis that the population multiple correlation equals zero with a power of 80%, alpha = 5% and a medium effect size for the regression analysis (R2=0.13).[46] In Phase II, the number of predictors reflects the casemix variables, and needs to be clinically relevant[47] to ensure a meaningful case-mix classification. We expect the final casemix classification to contain 5-8 casemix variables, based on the Australian experience.[13] We have estimated that data from 114 (50 + (8x8)) episodes of care per setting (hospice, hospital, community) are needed. Assuming an average of three episodes per participant[15] in this longitudinal phase, this represents at least 38 participants per setting. Allowing for 50% attrition, we estimate needing a total of 228 participants, which will provide about 684 patient episodes. The sample size will be re-calculated and inflated using the design effect based on intraclass correlation which will be estimated through interim analyses. A total of 300 participants across settings is needed, including extra 25% participants allowing for intraclass correlation.

Missing data and data handling

Data will be collected prospectively and recorded on an electronic database. Any data transferred from the sites to the central study database will be carried out under the NHS Code of Practice on Confidentiality. Data will be cleaned and cross-checked using a number of internal checks (automated data validation on entry, independent cross-checking of a 5% sample of data from each site, and range checking across all data types) to track errors and inconsistencies and amend where possible. Checked data will be transferred to statistical software (Stata SE v12) for analysis. Missing Value Analysis will be used to quantify missing values and understand the reason for missing data: drop-out of participants, errors in data entry, and missing with no identifiable reasons. We will adopt multiple imputation technique based on Markov Chain Monte Carlo (MCMC) techniques if the percentage of observed missing values in independent variables exceeds 10% provided values are

For peer review only - http://bmjopen.bmj.com/site/about/guidelines.xhtml

missing at random, and carry out sensitivity analysis for effects on casemix classification, predictive validity, and misclassification, where feasible.

Statistical analysis methods

We will use descriptive statistics including frequencies, mean and standard deviation; and median and interquartile ranges as appropriate to describe the sample characteristics, and number and length of episodes of care. Trajectory of outcomes and costs will be described, comparing patterns in cancer and non-cancer patients, and contrasting those with no unmet needs and those with unmet needs. We will compare our data with existing phase and episode data from the Australian Palliative Care Outcome Collaboration.[48] In the Australian classification, five casemix criteria (phase of illness, problem severity, functional status and dependency, age, model of care) were found to be most strongly predictive of resource use. If our analyses demonstrate the same five criteria as most predictive of resource use, the Australian classification or a refinement of it will be adopted. If there is disparity, then the data on individual casemix criteria will be used to develop a new casemix classification, using a recursive partitioning approach – specifically classification and regression trees (CART)[49, 50] compared to multivariable regression (Figure 1).

Figure 1 Data analysis flowchart here

Qualitative analysis methods

Audio-recordings will be transcribed verbatim and checked for accuracy and entered into NVivo 10. Data will be independently coded and analysed by two members of the research team. We will adopt a similar approach to Pinnock,[51] undertaking a thematic and narrative analysis of interviews, exploring how perspectives evolve over time, with detailed attention to patient and family perspectives on experience of care in each setting and transitions, including potential interventions (and hence cost levers or other triggers) to influence changes in settings of care.

Model development

The distribution of casemix criteria (cost predictors) in the participant population will be described based on Phase of Illness, functional status and problem severity, using parametric or non-parametric statistics, as appropriate. In order to reduce the number of cost predictors, we will initially use univariate analysis to explore the association and strength of association between casemix criteria and cost of the episode of care (quantifying cost by per diem cost, per phase cost

For peer review only - http://bmjopen.bmj.com/site/about/guidelines.xhtml

and total cost of episode). We will use generalised linear model (multivariate regression model) to select variables that will then be applied in a hierarchical manner to form a branching classification in which each cost driver is incorporated only once.

We will select variables according to how much of the predictive error (R-square) each variable predicts and the related p-value. After each bootstrap iteration we will rank the variables according to these two criteria and remove the weakest (non-significant) predictor. Variables shown in Table 2 and 3 and the newly created categorical variable representing 'model of care' will be initially entered into the models and then non-significant predictors removed at each iteration using a Bootstrap sub-sampling strategy. We will create dummy variables to enable each level of Phase of Illness and Model of Care (categorical variables) to be assessed individually. Internal validation will be conducted by using such methods as bootstrapping or cross-validation to quantify any optimism in the predictive performance (calibration and discrimination) of the developed classification.

Model specification

The casemix classification for each outcome across three settings will be presented including all regression coefficients and model intercept (Figure 2). How to use the classification will be explained. Understanding how casemix variables predict cost of the episode of care enables a robust casemix classification to be developed. But in order for casemix adjustment to occur, there also needs to be a good understanding of how the casemix variables impact on clinical outcomes, as well as resource use. This casemix adjustment methodology is described in the recently published Department of Health document,[52] which recommends that significant casemix variables are identified, and that the size of their relationship with clinical outcomes is determined in advance.

Multi-level modelling will be undertaken to ensure both of these steps to be delivered in this study by determining (1) which casemix criteria are most strongly associated with clinical outcomes, allowing for clustering at the level of 'participant' (where episodes occur in the same individual), at the level of 'model of care' (where care is received according to similar models), and at the level of 'site' (where care is delivered in a similar way within site), and (2) which casemix criteria are the strongest cost predictor (including per diem cost, per phase cost, and total episode cost).

Figure 2 Casemix classification specification here

For peer review only - http://bmjopen.bmj.com/site/about/guidelines.xhtml

Model performance

The performance of the casemix classification will be assessed in both the development and validation datasets. Classification measures including sensitivity, specificity, predictive values, and net reclassification improvement will be reported and cut points selected a-priori. We will primarily focus on discrimination for the classification development phase, and assess both discrimination and calibration for the validation phase.[53] Discrimination of the classification will be measured using the concordance statistic and confidence intervals (c-statistic).[54] Calibration of the classification will be measured using calibration plot and Hosmer-Lemeshow goodness-of-fit test.[55]

Development vs. validation

Data will be collected from patients, caregivers, and clinicians during the validation phase to compare patient-reported problem severity i.e. 'felt' need collected in validation phase only (as measured by the patient-reported IPOS) and professional-reported problem severity i.e. 'normative' need (as measured by the clinician-reported IPOS, Phase of Illness, AKPS), by reporting correlation across levels of complexity and across conditions. We will assess how professional-reported measures relate to patient-reported outcomes across different levels of complexity (i.e. in relation to casemix classes), in order to determine optimal outcome measures and quality indicators.

We will compare data on resource use from two sources: i) clinician-completed brief PRUS, and ii) patients and family-completed Client Service Receipt Inventory, to better understand how the PRUS maps to patients' receipt of resources, and evaluate which generic services are utilised across different providers, regions, and the context of different models of specialist palliative care.

Ethical considerations

The study protocol and documents (e.g., the participant information sheet, consent and declaration form) have been approved by the NHS Health Research Authority London – Camberwell St Giles Research Ethics committee (15/LO/0887) for the development phase, and NHS Health Research Authority London – Bromley Research Ethics committee (16/LO/1021) for the validation phase.

All eligible participants are fully informed before consent is sought by local research team or research nurses or project research team through the information sheets and verbal explanation on the aims and methods of the study and procedures that might be involved. Participants can withdraw at any time up to analysis of their data, without giving any reason. It is possible that

For peer review only - http://bmjopen.bmj.com/site/about/guidelines.xhtml

participants may become distressed or raise issues during this study which raise concerns or warrant a change in their medical management, but we do not expect the questionnaires will themselves cause distress so much as uncover pre-existing distress which has not been acknowledged or recognised. Should this be the case, then our distress protocol will be followed. We anticipate distress will be infrequent, given the general nature of the questionnaires. It is likely that any distress will reflect advanced disease and experiences of care, and not the questionnaires themselves. All of the study team members have completed Good Clinical Practice training, and specific training on addressing distress in palliative care.

Our existing Patient/Public Advisory Group and extended Consumer Panel have been and will continue to be consulted throughout the study to ensure that the study is carried out in an ethical and respectful way, and has the highest possible relevance and benefit to patients and families. A Project Steering Committee meets six monthly to monitor recruitment, review the detailed progress of the study, and make recommendations for overall direction and strategy.

Dissemination

This study will lead to patient benefit through improved matching of resources to needs at individual patient-level and will better enable the NHS to deliver high quality, patient-centred palliative care in last year of life. The results of the study will be published in peer-reviewed publications and will also be presented at national and international conferences.

For peer review only

For peer review only - http://bmjopen.bmj.com/site/about/guidelines.xhtml

Acknowledgements

This article presents independent research C-CHANGE funded by the National Institute for Health Research (NIHR) under the Programme Grants for Applied Research scheme (RP-PG-1210-12015) and the NIHR CLAHRC funding scheme, through the Collaboration for Leadership in Applied Health Research and Care (CLAHRC) in South London. CLAHRC South London is part of the NIHR and is a partnership between King's Health Partners, St George's, University of London, and St George's Healthcare NHS Trust. The C-CHANGE research team acknowledges the support of the National Institute of Health Research Clinical Research Network (NIHR CRN).

Contributors

PG drafted the study protocol with input from all authors. MD, JMD and AD provided statistical advice and support. AMF, SMO, CP, SP and PG contributed to the acquisition and interpretation of data for the work. IJH and FEM conceived the study and were in charge of overall direction and planning. KE provided experience from the Australian palliative care casemix development.[13] FEM is the Chief Investigator and designed the C-CHANGE research programme of which this is part. All authors provided critical feedback, helped revise the manuscript, and are accountable for the accuracy and integrity of all aspects of the work.

Funding

This work was supported by NIHR Programme Grants for Applied Research (Grant number RP-PG-1210-12015).

Disclaimer

The views and opinions expressed are those of the authors and do not necessarily reflect those of the NHS, the NIHR, MRC, CCF, NETSCC, the NIHR Programme Grants for Applied Research, or the Department of Health.

Competing interests

None declared.

Ethics approval

The NHS Health Research Authority (HRA) Approval.

For peer review only

For peer review only - http://bmjopen.bmj.com/site/about/guidelines.xhtml

References

1. Currow DC, Abernethy AP and Fazekas BS. Specialist palliative care needs of whole populations: a feasibility study using a novel approach. *Palliative Medicine*. 2004; 18: 239-47.
2. Fitch MI. Needs of patients living with advanced disease. *Canadian Oncology Nursing Journal* 2005; 15: 230-42.
3. Department of Health. Assessing health care needs. London: National Health Service Management Executive, 1991.
4. The Economist Intelligence Unit. The 2015 Quality of Death Index: Ranking palliaitve care across the world. 2015.
5. Sleeman KE, Davies JM, Verne J, Gao W and Higginson IJ. The changing demographics of inpatient hospice death: Population-based cross-sectional study in England, 1993–2012. *Palliative Medicine*. 2016; 30: 45-53.
6. Rosenwax L, Spilsbury K, McNamara BA and Semmens JB. A retrospective population based cohort study of access to specialist palliative care in the last year of life: who is still missing out a decade on? *BMC Palliative Care*. 2016; 15: 46.
7. Hughes-Hallett T, Craft A, Davies C, Mackay I and Nielsson T. Funding the right care and support for everyone: Creating a fair and transparent funding system; the final report of the palliative care funding review. *Palliative Care Funding Review*. 2011.
8. Lancaster H, Finlay I, Downman M and Dumas J. Commissioning of specialist palliative care services in England. *BMJ Supportive & Palliative Care*. 2017.
9. Etkind SN, Bone AE, Gomes B, et al. How many people will need palliative care in 2040? Past trends, future projections and implications for services. *BMC medicine*. 2017; 15: 102.
10. Lee LA, Eagar KM and Smith MC. Subacute and non-acute casemix in Australia. *The Medical Journal of Australia*. 1998; 169: 22-5.
11. Eagar K, Cromwell D, Kennedy C and Lee L. Classifying sub-acute and non-acute patients: Results of the New South Wales Casemix Area Network study. *Australian Health Review*. 1997; 20: 26-42.
12. Turner-Stokes L, Sutch S, Dredge R and Eagar K. International casemix and funding models: lessons for rehabilitation. *Clinical rehabilitation*. 2011; 26: 195-208.
13. Eagar K, Gordon R, Hodkinson A, et al. The Australian National Sub-Acute and Non-Acute Patient Classification (AN-SNAP): report of the National Sub-Acute and Non-Acute Casemix Classification Study. Centre for Health Service Development, University of Wollongong1997.
14. Eagar K, Green J and Gordon R. An Australian casemix classification for palliative care: technical development and results. *Palliative Medicine*. 2004; 18: 217-26.
15. Eagar K, Watters P, Currow DC, Aoun SM and Yates P. The Australian Palliative Care Outcomes Collaboration (PCOC)--measuring the quality and outcomes of palliative care on a routine basis. *Australian Health Review*. 2010; 34: 186-92.
16. Eagar K, Gordon R, Green J and Smith M. An Australian casemix classification for palliative care: lessons and policy implications of a national study. *Palliative Medicine*. 2004; 18: 227-33.
17. Collins GS, Reitsma JB, Altman DG and Moons KM. Transparent reporting of a multivariable prediction model for individual prognosis or diagnosis (tripod): The tripod statement. *Annals of Internal Medicine*. 2015; 162: 55-63.
18. Hinds J. *National Survey of Patient Activity Data for Specialist Palliative Care Services: Minimum Data Set (MDS) Full Report for the Year 2014-2015*. London: The National Council for Palliative Care, 2016.
19. Castro FG, Kellison JG, Boyd SJ and Kopak A. A methodology for conducting integrative mixed methods research and data analyses. *Journal of Mixed Methods Research*. 2010; 4: 342-60.
20. Hjermstad M, Loge JH and Kaasa S. Methods for assessment of cognitive failure and delirium in palliative care patients: implications for practice and research. *Palliative Medicine*. 2004; 18: 494-506.

For peer review only - http://bmjopen.bmj.com/site/about/guidelines.xhtml

21. Radbruch L, Sabatowski R, Loick G, et al. Cognitive impairment and its influence on pain and symptom assessment in a palliative care unit: development of a Minimal Documentation System. *Palliative Medicine*. 2000; 14: 266-76.
22. Higginson IJ, McCrone P, Hart SR, Burman R, Silber E and Edmonds PM. Is Short-Term Palliative Care Cost-Effective in Multiple Sclerosis? A Randomized Phase II Trial. *Journal of Pain and Symptom Management*. 2009; 38: 816-26.
23. Bausewein C, Booth S, Gysels M, Kühnbach R, Haberland B and Higginson IJ. Individual breathlessness trajectories do not match summary trajectories in advanced cancer and chronic obstructive pulmonary disease: results from a longitudinal study. *Palliative Medicine*. 2010; 24: 777-86.
24. Buller T. Advance consent, critical interests and dementia research. *Journal of Medical Ethics*. 2015; 41: 701-7.
25. Mather H, Guo P, Firth A, et al. Phase of Illness in palliative care: Cross-sectional analysis of clinical data from community, hospital and hospice patients. *Palliative Medicine*. DOI: 10.1177/0269216317727157.
26. Masso M, Allingham SF, Banfield M, et al. Palliative Care Phase: Inter-rater reliability and acceptability in a national study. *Palliative Medicine*. 2015; 29: 22-30.
27. Coventry PA, Grande GE, Richards DA and Todd CJ. Prediction of appropriate timing of palliative care for older adults with non-malignant life-threatening disease: a systematic review. *Age and Ageing*. 2005; 34: 218-27.
28. Franks PJ, Salisbury C, Bosanquet N, et al. The level of need for palliative care: a systematic review of the literature. *Palliative Medicine*. 2000; 14: 93-104.
29. Luddington L, Cox S, Higginson I and Livesley B. The need for palliative care for patients with non-cancer diseases: a review of the evidence. *International Journal of Palliative Nursing*. 2001; 7: 221-6.
30. Tebbit P. *Population-based Needs Assessment for Palliaitve and End of Life Care*. London: National Council for Palliative Care, 2008.
31. Drummond M, Weatherly H and Ferguson B. *Economic evaluation of health interventions*. 2008.
32. Kirch W. *Encyclopedia of Public Health*. Dordrecht: Springer Netherlands, 2008, p.267.
33. Curtis L. *Unit costs of health and social care 2014*. Canterbury: Personal Social Services Research Unit, University of Kent, 2014.
34. *Joint Formulary Committe. British National Formulary (BNF) 68*. London: BMJ Publishing Group Ltd and Royal Pharmaceutical Society, 2014.
35. Department of Health. NHS reference costs 2013 to 2014. 2014.
36. Wolff J, McCrone P, Patel A, Auber G and Reinhard T. A time study of physicians' work in a German University Eye Hospital to estimate unit costs. *PLOS ONE*. 2015; 10: e0121910.
37. Sutherland JM. Pricing hospital care: Global budgets and marginal pricing strategies. *Health Policy*. 2015; (in press).
38. Tan SS, Bakker J, Hoogendoorn ME, et al. Direct cost analysis of Intensive Care Unit stay in four European countries: Applying a standardized costing methodology. *Value in Health*. 2012; 15: 81-6.
39. Department of Health. NHS costing manual. 2012.
40. Abernethy AP, Shelby-James T, Fazekas BS, Woods D and Currow DC. The Australia-modified Karnofsky Performance Status (AKPS) scale: a revised scale for contemporary palliative care clinical practice [ISRCTN81117481]. *BMC Palliative Care*. 2005; 4: 7.
41. Collin C, Wade DT, Davies S and Horne V. The Barthel ADL Index: a reliability study. *International disability studies*. 1988; 10: 61-3.
42. King J, Gibbons E, Graham C and Walsh J. Developing measures of people's self-reported experiences of integrated care. *Picker Institute Europe and University of Oxford*. 2013.

For peer review only - http://bmjopen.bmj.com/site/about/guidelines.xhtml

43. Koffman J, Higginson IJ, Hall S, Riley J, McCrone P and Gomes B. Bereaved relatives' views about participating in cancer research. *Palliative Medicine*. 2012; 26: 379-83.
44. Addington-Hall J and McPherson C. After-death interviews with surrogates/bereaved family members: Some issues of validity. *Journal of Pain and Symptom Management*. 2001; 22: 784-90.
45. Costantini M, Beccaro M and Merlo F. The last three months of life of Italian cancer patients. Methods, sample characteristics and response rate of the Italian Survey of the Dying of Cancer (ISDOC). *Palliative Medicine*. 2005; 19: 628-38.
46. VanVoorhis CW and Morgan BL. Understanding power and rules of thumb for determining sample sizes. *Tutorials in Quantitative Methods for Psychology*. 2007; 3: 43-50.
47. Ogundimu EO, Altman DG and Collins GS. Adequate sample size for developing prediction models is not simply related to events per variable. *Journal of clinical epidemiology*. 2016; 76: 175-82.
48. Currow DC, Allingham S, Yates P, Johnson C, Clark K and Eagar K. Improving national hospice/palliative care service symptom outcomes systematically through point-of-care data collection, structured feedback and benchmarking. *Supportive care in cancer : official journal of the Multinational Association of Supportive Care in Cancer*. 2015; 23: 307-15.
49. Gao J, Johnston GM, Lavergne MR and McIntyre P. Identifying population groups with low palliative care program enrolment using classification and regression tree analysis. *Journal of palliative care*. 2011; 27: 98-106.
50. Lemon SC, Roy J, Clark MA, Friedmann PD and Rakowski W. Classification and regression tree analysis in public health: Methodological review and comparison with logistic regression. *ann behav med*. 2003; 26: 172-81.
51. Pinnock H, Kendall M, Murray SA, et al. Living and dying with severe chronic obstructive pulmonary disease: multi-perspective longitudinal qualitative study. *BMJ*. 2011; 342.
52. Department of Health. Patient reported outcome measures (PROMs) in England: the case-mix adjustment methodology. 2012.
53. Steyerberg EW, Vickers AJ, Cook NR, et al. Assessing the performance of prediction models: a framework for some traditional and novel measures. *Epidemiology (Cambridge, Mass)*. 2010; 21: 128-38.
54. Cook NR. Statistical Evaluation of Prognostic versus Diagnostic Models: Beyond the ROC Curve. *Clinical Chemistry*. 2007; 54: 17.
55. Austin PC and Steyerberg EW. Graphical assessment of internal and external calibration of logistic regression models by using loess smoothers. *Statistics in Medicine*. 2014; 33: 517-35.

For peer review only

For peer review only - http://bmjopen.bmj.com/site/about/guidelines.xhtml

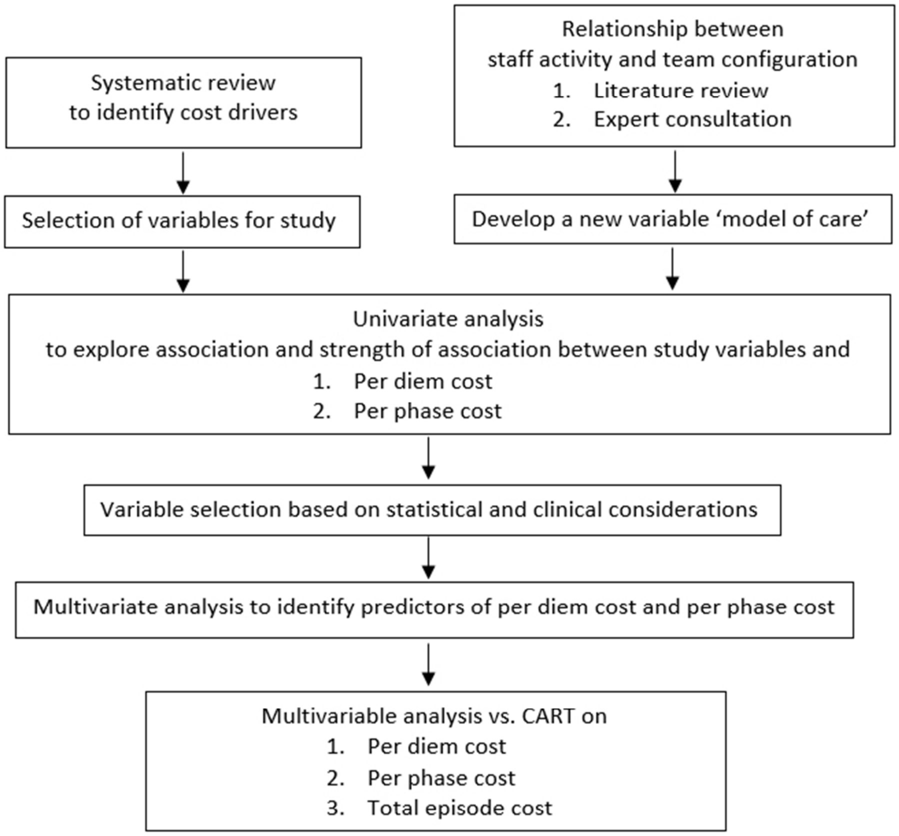

Figure 1 Data analysis flowchart

Figure 1 Data analysis flowchart

61x56mm (300 x 300 DPI)

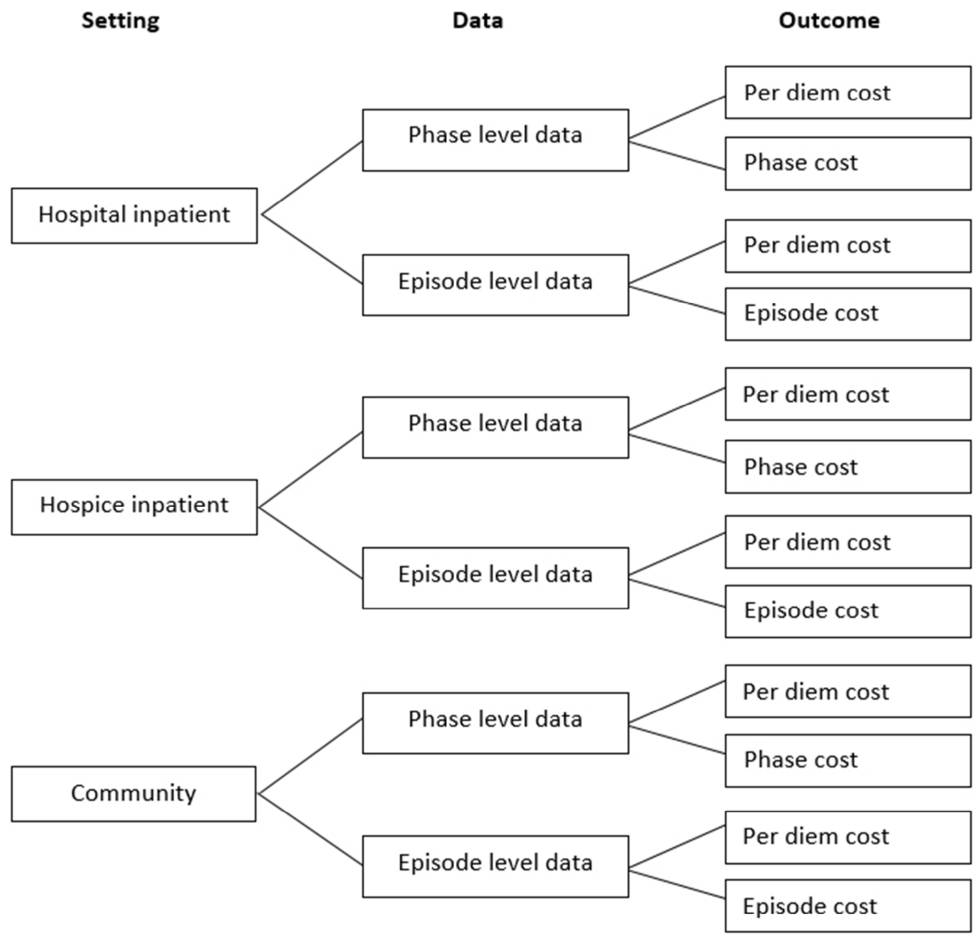

Figure 2 Casemix classification specification

Figure 2 Casemix classification specification

58x59mm (300 x 300 DPI)

TRIPOD Checklist: Prediction Model Development and Validation

Section/Topic	Item		Checklist Item	Page
**Title and abstract**				
Title	1	D;V	Identify the study as developing and/or validating a multivariable prediction model, the target population, and the outcome to be predicted.	1
Abstract	2	D;V	Provide a summary of objectives, study design, setting, participants, sample size, predictors, outcome, statistical analysis, results, and conclusions.	2
**Introduction**				
Background and objectives	3a	D;V	Explain the medical context (including whether diagnostic or prognostic) and rationale for developing or validating the multivariable prediction model, including references to existing models.	4-5
	3b	D;V	Specify the objectives, including whether the study describes the development or validation of the model or both.	4-5
**Methods**				
Source of data	4a	D;V	Describe the study design or source of data (e.g., randomized trial, cohort, or registry data), separately for the development and validation data sets, if applicable.	5-6
	4b	D;V	Specify the key study dates, including start of accrual; end of accrual; and, if applicable, end of follow-up.	5-6
Participants	5a	D;V	Specify key elements of the study setting (e.g., primary care, secondary care, general population) including number and location of centres.	6-7
	5b	D;V	Describe eligibility criteria for participants.	6-7
	5c	D;V	Give details of treatments received, if relevant.	6-7
Outcome	6a	D;V	Clearly define the outcome that is predicted by the prediction model, including how and when assessed.	7-9
	6b	D;V	Report any actions to blind assessment of the outcome to be predicted.	N/A
Predictors	7a	D;V	Clearly define all predictors used in developing the multivariable prediction model, including how and when they were measured.	9-11
	7b	D;V	Report any actions to blind assessment of predictors for the outcome and other predictors.	N/A
Sample size	8	D;V	Explain how the study size was arrived at.	12
Missing data	9	D;V	Describe how missing data were handled (e.g., complete-case analysis, single imputation, multiple imputation) with details of any imputation method.	12
Statistical analysis methods	10a	D	Describe how predictors were handled in the analyses.	13
	10b	D	Specify type of model, all model-building procedures (including any predictor selection), and method for internal validation.	13-15
	10c	V	For validation, describe how the predictions were calculated.	13-15
	10d	D;V	Specify all measures used to assess model performance and, if relevant, to compare multiple models.	13-15
	10e	V	Describe any model updating (e.g., recalibration) arising from the validation, if done.	13-15
Risk groups	11	D;V	Provide details on how risk groups were created, if done.	13-15
Development vs. validation	12	V	For validation, identify any differences from the development data in setting, eligibility criteria, outcome, and predictors.	15
**Results**				
Participants	13a	D;V	Describe the flow of participants through the study, including the number of participants with and without the outcome and, if applicable, a summary of the follow-up time. A diagram may be helpful.	N/A
	13b	D;V	Describe the characteristics of the participants (basic demographics, clinical features, available predictors), including the number of participants with missing data for predictors and outcome.	N/A
	13c	V	For validation, show a comparison with the development data of the distribution of important variables (demographics, predictors and outcome).	N/A
Model development	14a	D	Specify the number of participants and outcome events in each analysis.	N/A
	14b	D	If done, report the unadjusted association between each candidate predictor and outcome.	N/A
Model specification	15a	D	Present the full prediction model to allow predictions for individuals (i.e., all regression coefficients, and model intercept or baseline survival at a given time point).	N/A
	15b	D	Explain how to use the prediction model.	N/A
Model performance	16	D;V	Report performance measures (with CIs) for the prediction model.	N/A
Model-updating	17	V	If done, report the results from any model updating (i.e., model specification, model performance).	N/A
**Discussion**				
Limitations	18	D;V	Discuss any limitations of the study (such as nonrepresentative sample, few events per predictor, missing data).	12
Interpretation	19a	V	For validation, discuss the results with reference to performance in the development data, and any other validation data.	N/A
	19b	D;V	Give an overall interpretation of the results, considering objectives, limitations, results from similar studies, and other relevant evidence.	N/A
Implications	20	D;V	Discuss the potential clinical use of the model and implications for future research.	15-16
**Other information**				
Supplementary information	21	D;V	Provide information about the availability of supplementary resources, such as study protocol, Web calculator, and data sets.	N/A
Funding	22	D;V	Give the source of funding and the role of the funders for the present study.	17

*Items relevant only to the development of a prediction model are denoted by D, items relating solely to a validation of a prediction model are denoted by V, and items relating to both are denoted D;V. We recommend using the TRIPOD Checklist in conjunction with the TRIPOD Explanation and Elaboration document.

For peer review only - http://bmjopen.bmj.com/site/about/guidelines.xhtml
